# Deep learning and hybrid architectures for atypical and complex bone fracture diagnosis: a systematic review of performance and clinical validity

**DOI:** 10.3389/frai.2026.1909177

**Published:** 2026-08-10

**Authors:** Fatma Atitallah, Johannes C. Ayena, Assem Thabet, Neila Mezghani

**Affiliations:** 1 Research Laboratory MACS, National Engineering School of Gabes, University of Gabes, Gabes, Tunisia; 2 Applied Artificial Intelligence Institute (I2A), TELUQ University, Montreal, QC, Canada; 3 Open Innovation Laboratory in Health Technologies, CHUM Research Center, Montreal, QC, Canada

**Keywords:** AI, atypical fractures, complex fractures, detection, machine learning, medical imaging

## Abstract

Artificial intelligence (AI) is reshaping fracture diagnosis in medical imaging. Despite these advances, accurately identifying atypical fractures (such as stress or pathological fractures) and complex fractures (including comminuted and pelvic fractures) remains a significant clinical challenge. This systematic review evaluates the current evidence on AI models, including advanced architectures, for detecting, classifying, and segmenting atypical and complex bone fractures in humans. A total of 40 studies published between 2015 and 2026 met the predefined inclusion criteria. Eligible studies used real-world imaging modalities (X-ray, CT, or MRI), focused on atypical or complex fractures, employed AI-based approaches with expert-validated reference standards, and reported quantitative performance metrics. Studies based exclusively on synthetic data, restricted to simple fractures, or lacking adequate validation were excluded. Advanced AI models, including hybrid frameworks such as 3D U-Net variants and DeepLabV3+MobileNetV3, were associated with improved performance in several studies, particularly for identifying subtle and multi-fragment fractures. However, substantial heterogeneity in study design, datasets, validation strategies, and evaluation metrics limits direct comparisons across models. Hybrid systems, particularly CNN-based architectures combined with level-set methods or multi-network pipelines, also appeared effective in capturing complex fracture patterns in several studies, although this observation is based on a limited and heterogeneous body of evidence. Overall, the available evidence suggests that advanced AI models have considerable potential to improve the detection, classification, and segmentation of atypical and complex fractures. Nevertheless, the predominance of single-center studies, the limited use of external or prospective validation, and methodological heterogeneity indicate that further standardized, multicenter clinical validation is required before these models can be widely implemented in routine clinical practice.

## 1 Introduction

In recent years, Artificial Intelligence has emerged as a powerful tool in medical imaging (Meena and Roy, 2022; Wang et al., 2021), significantly improving the speed and accuracy of scan analysis. One notable application is the detection (Topol, 2019; Hosny et al., 2018) and segmentation of bone fractures (Litjens et al., 2017; Meena and Roy, 2022), which generally involves three functions: classification into specific categories, detection of fracture regions, and pixel-level segmentation of fracture contours (Su et al., 2023). Numerous studies (Meena and Roy, 2022; Kalmet et al., 2020; Kutbi, 2024) have emphasized the role of deep supervised learning models, such as CNNs, ResNet, and U-Net, in fracture detection and classification across X-ray, CT, and MRI, frequently achieving sensitivity and specificity close to 90% (Meena and Roy, 2022; Kutbi, 2024). However, atypical and complex fractures present significant challenges for both radiologists and AI systems due to their irregular patterns and subtle radiographic features (Rehman et al., 2019; Jain et al., 2024). Atypical fractures occur because of abnormal bone quality or abnormal loading rather than acute high-energy trauma. They include stress fractures and pathological fractures. Stress fractures result from repetitive loading or normal physiological stress applied to weakened bone, whereas pathological fractures occur in bone affected by tumors or metastases. These fractures are often difficult to detect because initial radiographs may appear normal or inconclusive, making accurate diagnosis essential for appropriate clinical management (Fayad et al., 2005). In addition, atypical fractures, such as fragility fractures, are frequently subtle, occur in unusual locations, and are therefore easily overlooked during the initial assessment (Zdolsek et al., 2021; Oh et al., 2021; Khan et al., 2024).

Complex fractures involve anatomically challenging regions, such as the pelvis and acetabulum, or multiple bone fragments, as seen in comminuted fractures. Their three-dimensional geometry complicates fracture classification and accurate delineation of fracture lines and fragment relationships. Consequently, CT, particularly with 3D reconstructions, is often required for reliable characterization (Scheinfeld et al., 2015). These characteristics also make complex fractures more challenging for automated segmentation, especially in pelvic injuries where precise delineation is critical for surgical planning (Zeng et al., 2024; Liu Y. et al., 2025; Zhou et al., 2024). Given these challenges, AI approaches have been increasingly explored to improve fracture detection, classification, and localization. Understanding the main AI tasks, particularly image classification and segmentation, is therefore essential for evaluating their application in complex fracture diagnosis. Image classification assigns an image to a predefined category based on visual content, typically via CNNs that learn hierarchical feature representations (Chen et al., 2021), while image segmentation partitions an image into meaningful regions based on visual characteristics such as color, texture, or intensity (Yu et al., 2023). Traditionally, fracture diagnosis has relied on expert radiologists (Wood et al., 2012) or classical CNN-based models (Meena and Roy, 2022; Kalmet et al., 2020); however, manual annotation is time-consuming and subjective, and conventional models often fail to generalize across diverse fracture types (Litjens et al., 2017; Kalmet et al., 2020). This limitation has motivated recent research on advanced architectures incorporating attention mechanisms, which can better capture global context and fine-grained fracture characteristics (Zhou et al., 2024; Lee et al., 2024).

X-ray remains the most widely used modality in clinical practice owing to its affordability, availability, and rapid acquisition (Aldhyani et al., 2025; Abedeen et al., 2023; Liu et al., 2020; Zhang et al., 2021). However, its limitations become apparent for subtle fissures, comminuted fragments, or deeply situated injuries (Jarraya et al., 2013). CT, by contrast, provides higher spatial resolution and three-dimensional reconstruction, allowing more detailed visualization of complex bone structures and improved detection of fractures in anatomically challenging regions such as the pelvis, ribs, and spine (Zhou Q.-Q. et al., 2022; Dreizin et al., 2021; Zakharov et al., 2023). MRI offers more detailed visualization but remains rarely used in routine workflows due to cost, limited accessibility, and under-representation in public datasets (Wang et al., 2021; Jarraya et al., 2013; Litjens et al., 2017; Emon et al., 2022). These limitations raise an important question regarding whether current AI approaches can reliably support the detection and segmentation of complex fractures, particularly when subtle or irregular patterns are involved. Existing reviews illustrate important gaps in this field. Meena and Roy (2022) reviewed supervised deep learning approaches in radiography, showing promising results for mild to moderate fractures but not addressing atypical fractures or multimodal imaging. Kutbi (2024) extended this scope to multiple imaging modalities and attention-based models but highlighted the limited availability of comprehensive clinical validation. Kalmet et al. (2020) emphasized the need for validation using heterogeneous, real-world datasets, noting that most studies focused on relatively simple or moderately complex fractures. More specifically, Pham et al. (2024), published in *Frontiers in Artificial Intelligence*, examined facial trauma fracture diagnosis using CNN, YOLO, U-Net, and RetinaNet architectures; however, their review was restricted to the craniofacial region, did not address atypical or multi-fragment/pelvic fractures, and covered literature only up to early 2023.

Despite growing evidence on AI-based fracture detection and segmentation, existing reviews have several limitations. Most have focused on general fracture presentations, single anatomical regions, or conventional CNN architectures. In addition, limited attention has been given to atypical and complex fracture patterns, while the use of formal quality-assessment frameworks has remained inconsistent. Table 1 summarizes these prior reviews by year, fracture scope, imaging modalities, AI architectures, quality assessment, and the specific gap addressed by the present work.

**Table 1:** Comparison of previous reviews and the present review on AI-based fracture analysis.

Prior review	Year	Fracture scope	Modalities	AI models covered	Quality assessment	Gap addressed
Meena and Roy (2022)	2022	Overview of upper limb, lower limb, and vertebral fractures	X-ray (predominant), CT, and MRI	CNN, ResNet, VGGNet, Inception, DenseNet, U-Net, Attention-guided GAN, Self-supervised learning	None/Not reported	Provides a comprehensive mapping of deep learning approaches in bone radiology. Highlights the lack of standardization in data annotation, the risk of overfitting, and the ethical, legal, and technical barriers limiting clinical deployment
Kutbi (2024)	2024	Multi-site (upper limbs, lower limbs, skull, mandible, thorax, vertebrae, dental roots)	X-ray, CT/3D CT	CNNs (AlexNet, VGG, Inception, ResNet, DenseNet-121), RNN, visual attention mechanisms, probabilistic networks, and theoretical introduction of LLMs (GPT-3/4)	None/Not reported	Addresses the lack of analysis on AI's clinical impact (efficiency, cost, accessibility) and its integration with advanced imaging (3D CT) and emerging technologies like LLMs and Generative AI.
Kalmet et al. (2020)	2020	General fracture detection and classification (with focus on wrist, proximal humerus, and osteoporotic vertebral fractures)	Radiographs and CT examinations	VGG16, Inception V3, Xception, ResNet (e.g., ResNet-152), U-net, Mask-RCNN, Faster-RCNN, and CNN+RNN hybrids	None/Not reported	Addresses the radiology workforce shortage and increasing workload strain by exploring deep learning solutions, while specifically highlighting the scarcity of fracture detection studies on CT scans compared to radiographs.
Pham et al. (2024)	2024	Maxillofacial/Facial trauma fractures and skull/cranial fractures	CT scans and X-ray radiography	CNNs (DenseNet-169, ResNet-152), Object detection (YOLOv4, YOLOv5, Faster R-CNN), Segmentation (ResUNet++), RNNs, and GANs/VAEs	None/Not reported	Identifies the lack of AI research dedicated specifically to complex 3D facial/midfacial bone frameworks, while highlighting human diagnostic challenges like overlapping bone fragments and soft-tissue swelling.
Present review	2026	Broad multi-site scope: vertebral, pelvic, rib, femoral, tibial, and craniofacial/complex fractures (including atypical patterns).	CT scans (including 3D CT) and X-ray radiography (multimodal fusion).	Advanced hybrid models and Transformer-based architectures (e.g., UNETR, GCViT, nnU-Net, RetinaNet, YOLOv12, and FracNet).	QUADAS-2 framework (bias across 4 domains, plus AI metrics: dataset quality, validation robustness, and clinical usability).	This review addresses the limitations of previous studies by specifically covering atypical and complex fractures (vertebral, pelvic, rib, femoral, tibial, and craniofacial fractures) across multiple imaging modalities and advanced AI architectures (including transformers and hybrid models). It provides an updated literature analysis up to 2026 and performs a rigorous evaluation using an AI-adapted QUADAS-2 framework to assess clinical validity.

Several recent reviews on AI-based fracture detection were identified during the literature search; however, they were not included in the direct comparison table because their objectives and scopes differed from those of the present review. For example, Jung et al. (2024) was excluded because its main objective was to evaluate pooled diagnostic performance measures (sensitivity and specificity) through meta-analysis across different imaging modalities and datasets, rather than to provide a comprehensive mapping of anatomical fracture sites, AI architectures, and clinical applicability. Similarly, van den Broek et al. (2024) was excluded because it focused exclusively on rib fracture detection using CT and radiography, representing a single anatomical site and a limited imaging scope, which reduced its comparability with the broader multi-site and multimodal perspective of this review.

To address the limitations identified in previous reviews, the present systematic review focuses specifically on atypical and complex fractures across multiple anatomical regions. It extends the anatomical scope to vertebral, pelvic, rib, femoral, tibial, and craniofacial fractures, incorporates recent studies up to 2026, and examines advanced AI architectures, including transformer-based and hybrid approaches. In addition, an AI-adapted QUADAS-2 framework is applied to assess methodological quality, validation strategies, and clinical readiness, providing a comprehensive evaluation of clinically challenging fracture cases, including occult and atypical fractures.

The general objective of this review is therefore to assess and compare the performance of advanced architectures, such as transformer-based and hybrid models, in the detection and segmentation of complex and atypical bone fractures across imaging modalities including CT and X-ray. While X-rays dominate both clinical settings and public datasets, they are not always the most reliable option for subtle or complex fractures; several studies instead highlight the value of 3D CT (Moon et al., 2024; Ukai et al., 2021) for more accurate detection, segmentation, and classification, underscoring the need to evaluate each modality's advantages and the potential benefits of combining them (Rahman, 2024; Meadi et al., 2024).

To guide the scope and structure of this review, four research questions were formulated: (1) Which AI architectures (CNN, transformer, hybrid) are applied to atypical and complex fracture detection, classification, and segmentation? (2) How does reported performance vary by task, imaging modality, and fracture type? (3) What are the main methodological and validation gaps limiting generalizability? (4) What evidence currently supports progression toward clinical implementation?

## 2 Methods

### 2.1 Inclusion and exclusion criteria

To ensure a rigorous and transparent study selection process, the eligibility criteria were predefined and are summarized in Table 2.

**Table 2:** Inclusion and exclusion criteria for study selection.

Inclusion criteria	Exclusion criteria
Focused on complex or atypical fractures.	Addressed only simple or typical fractures.
Used real clinical imaging modalities (X-ray, CT, and MRI).	Relied solely on synthetic imaging data.
Employed AI techniques (CNNs, transformers, and hybrid models).	Lacked validated or reproducible outcomes.
Included comparisons with other AI systems or diagnostic standards.	Provided non-verifiable or incomplete data.
Used expert-annotated, non-synthetic datasets.	Systematic reviews, narrative reviews, and survey articles.
Reported validated performance metrics (Dice, IoU, sensitivity, and specificity).	—
Peer-reviewed original research articles and academic theses.	—
Published between February 2015 and March 2026 in English or French.	—

Because this review specifically focuses on complex and atypical fractures, additional operational definitions were established to ensure consistent classification and reproducibility during study selection, as presented in Table 3.

**Table 3:** Operational definitions of atypical and complex fractures, grounded in the studies included in this review.

Category	Operational definition	Included examples (with source)	Excluded examples
Atypical fracture	Fractures occurring in abnormal bone conditions (osteoporotic, pathological, stress, or insufficiency-related) or presenting subtle and unusual radiographic features that increase the risk of missed diagnosis, representing a distinct category from conventional stress fractures (Oh et al., 2021).	Atypical femoral fracture (AFF) vs. normal femoral fracture (NFF) (Zdolsek et al., 2021; Schilcher et al., 2024); osteoporotic/insufficiency fracture (Khan et al., 2024); neoplastic pathological fracture of the hip, differentiated from non-pathologic fracture (Kim et al., 2021); hairline fracture (Jain et al., 2024).	• Standard high-energy traumatic fractures on healthy bone (Blüthgen et al., 2020).
Complex fracture	Fractures characterized by multiple fragments, comminution, complex three-dimensional geometry, or intra-articular/pelvic ring involvement, resulting in increased challenges for accurate segmentation, classification, and surgical planning (Zeng et al., 2024).	Comminuted/multi-fragment pelvic fracture with Tile AO/OTA grading (Dreizin et al., 2021; Zeng et al., 2024; Chen et al., 2025); multi-fragment rib fracture (Zhou Q.-Q. et al., 2022; Jin et al., 2020); complex tibial plateau fracture with Schatzker classification (van der Gaast et al., 2025); multiple/multi-level vertebral compression fractures (Choi et al., 2023); fractures explicitly labeled as “complex” in the source study taxonomy (Turk et al., 2024).	• Simple long-bone diaphyseal fractures (Vironicka and Sathiaseelan, 2023). • Single-fragment closed fractures without displacement (Bae et al., 2021).

### 2.2 Search strategy

To systematically identify relevant studies, a structured search strategy was designed to capture a comprehensive body of literature spanning both clinical and technical domains. The search was conducted across four major databases: PubMed, IEEE Xplore, Google Scholar, and the Cochrane Library. These databases were selected to ensure broad multidisciplinary coverage, including biomedical research, clinical applications, artificial intelligence, and engineering studies. In particular, PubMed was used for its extensive biomedical literature, IEEE Xplore for its focus on engineering and technological advances, Google Scholar for its wide-ranging academic indexing, and the Cochrane Library for evidence-based medical research (Paez, 2017; Falagas et al., 2007).

The review was initiated by defining a key research question and establishing a predefined methodological framework based on the PICO approach and structured review concepts. This framework encompassed the research objectives, eligibility criteria, search strategy, study selection process, data extraction, and quality assessment procedures. Although the review protocol was not prospectively registered, the study was conducted in accordance with the PRISMA 2020 guidelines.

All databases were searched up to 15 March 2026, with the final literature search completed on that date. Titles and abstracts were first screened, followed by full-text assessment of potentially eligible studies according to predefined inclusion and exclusion criteria. Any disagreements between the reviewers were resolved through discussion and consensus. Inter-reviewer agreement was not formally calculated; however, all discrepancies were resolved prior to final study inclusion.

The search strategy was built around five key thematic concepts and refined using Boolean operators, such as AND and OR, to optimize query combinations. Both free-text keywords and controlled vocabulary terms, including MeSH where applicable, were used to enhance retrieval accuracy. In PubMed and the Cochrane Library, searches were performed in the title, abstract, and controlled vocabulary (MeSH) fields. In IEEE Xplore, searches were conducted within the title, abstract, and author keyword fields. Google Scholar does not support field-specific searching comparable to PubMed or IEEE Xplore; therefore, predefined keyword combinations were searched using the default interface, which retrieves records across the indexed metadata and full-text where available. The first 1,843 records ranked by relevance were screened. Duplicate records were removed before the screening process. This approach was adopted to maximize search sensitivity while minimizing the risk of missing potentially relevant studies.

The search was restricted to studies published between 15 February 2015 and 15 March 2026 to capture the rapid evolution of artificial intelligence in fracture analysis, encompassing the emergence of deep learning, hybrid architectures, and more recent transformer-based models.Although the search strategy covered publications in both English and French, only English-language publications were identified and met the eligibility criteria. Covidence software was used to manage study records, including duplicate removal and the screening workflow. To ensure reproducibility, the complete search strings for each database, including Boolean operators, field specifications, and controlled vocabulary terms where applicable, are provided in Supplementary Table 1.

### 2.3 Data synthesis approach

A quantitative meta-analysis was not performed because substantial clinical and methodological heterogeneity was observed across the included studies. This heterogeneity included differences in imaging modalities, fracture types, AI architectures, task definitions (classification, detection, segmentation, or hybrid approaches), and reported performance metrics (AUC, sensitivity, Dice, and IoU). In addition, inconsistent reporting standards and heterogeneous definitions of outcomes prevented statistically meaningful pooling of effect sizes.

Preliminary assessment indicated that any pooled estimate would likely be unreliable and potentially misleading due to between-study variability. Subgroup pooling based on shared performance metrics and imaging modalities was also considered; however, even within such restricted subsets, clinically non-equivalent tasks were observed (e.g., segmentation of vertebral fractures vs. multi-fragment pelvic fractures), violating the assumption of exchangeability required for random-effects meta-analysis. Furthermore, no sufficiently homogeneous subgroup with an adequate number of comparable studies was identified to support reliable quantitative synthesis.

Instead, a structured narrative synthesis was conducted following recommendations for systematic reviews of diagnostic AI studies with high between-study heterogeneity (Guni et al., 2024). This approach enabled a meaningful comparison of studies according to clinical task, imaging modality, fracture type, and validation level, while preserving the clinical and methodological context of each study.

### 2.4 Selection process

This systematic review was not prospectively registered, and no review protocol was published. The final literature search was conducted on 15 March 2026. A comprehensive literature search was conducted across four electronic databases, yielding 3,438 records: Google Scholar (*n =* 1,843), PubMed (*n =* 1,520), Cochrane Library (*n =* 47), and IEEE Xplore (*n =* 28). No additional studies were identified through manual searching, citation tracking, or gray literature sources. Following duplicate removal using Covidence software (Veritas Health Innovation, 2014), 2,557 unique records were screened based on titles and abstracts. Screening and eligibility assessment were performed independently by two reviewers using Covidence software and followed the PRISMA 2020 guidelines. Disagreements between reviewers were resolved through discussion until consensus was reached. Inter-reviewer agreement (e.g., Cohen's kappa) was not calculated, as all discrepancies were resolved through consensus-based discussion. At the title and abstract stage, 2,083 records were excluded because they did not meet the eligibility criteria defined in Table 2, including non-fracture topics, absence of AI-based methodology, non-human studies, or conference abstracts without full text. In accordance with PRISMA 2020 recommendations, detailed sub-categorization of exclusions is not required at this stage. The remaining 474 full-text articles were assessed for eligibility. Full-text screening was conducted in Covidence, which recorded explicit reasons for exclusion for each study. A total of 434 studies were excluded for the following reasons: wrong patient population (*n =* 128), wrong study design (*n =* 98), wrong intervention (*n =* 95), wrong outcomes (*n =* 65), wrong indication (*n =* 27), wrong setting (*n =* 13), wrong methodology or focus (*n =* 7), and wrong comparator (*n =* 1). No studies were classified as ongoing or awaiting classification at the end of the screening process. The exact Boolean search strategies used for each database are provided below to ensure full reproducibility.

**PubMed:** ("Fractures, Bone"[MeSH Terms] OR "atypical fracture*"[All Fields] OR "complex fracture*"[All Fields]) AND ("deep learning"[All Fields] OR "Artificial Intelligence"[MeSH Terms] OR "convolutional neural network*"[All Fields]) AND ("Dice score"[All Fields] OR sensitivity[All Fields] OR specificity[All Fields]) AND (English[Language] OR French[Language]) AND ("2015/02/15"[Date - Publication] : "2026/03/15"[Date - Publication])
**Google Scholar:** ("bone fracture" OR "skeletal fracture" OR "hip fracture" OR "femoral fracture" OR "atypical fracture*" OR "complex fracture*") AND ("deep learning" OR "machine learning" OR CNN OR "convolutional neural network") AND ("Dice score" OR sensitivity OR specificity OR "AUC ROC"); filters: 2015–2026, English/French, patents excluded, sorted by relevance (first 1843 records screened due to platform relevance ranking limitations)
**Cochrane Library:** (atypical NEXT fracture* OR complex NEXT fracture*) AND (fracture NEXT detection* OR deep NEXT learning*) AND (Dice NEXT score* OR sensitivity OR specificity OR AUC-ROC); restricted to 2015–2026 and English/French
**IEEE Xplore:** ("atypical fracture*" OR "complex fracture*" OR "bone stress injury") AND ("fracture detection" OR "deep learning" OR "transformer-based models") AND ("Dice score" OR sensitivity OR "AUC-ROC"); restricted to 2015–2026 and English/French

**Figure 1:**
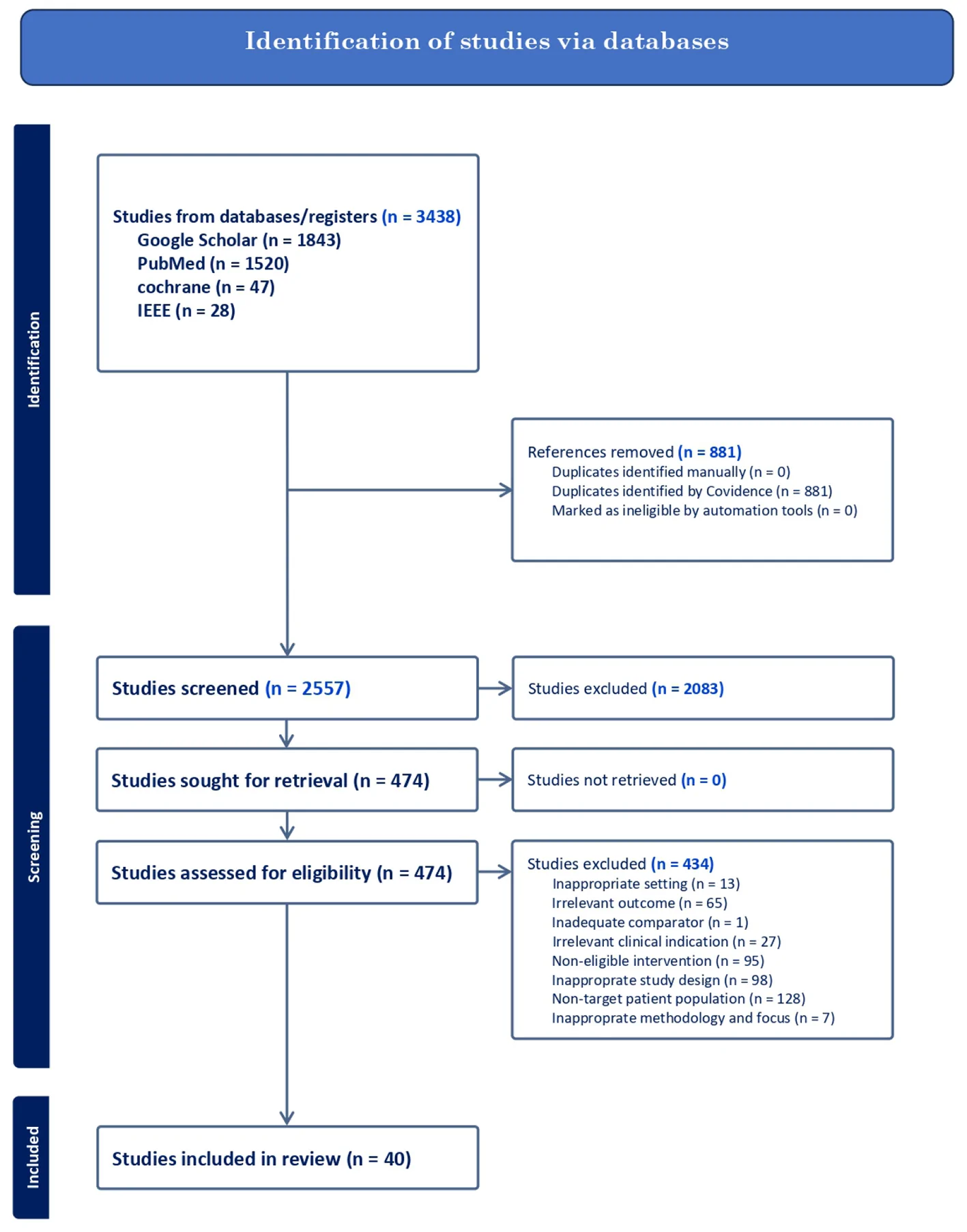
Preferred Reporting Items for Systematic Reviews and Meta-Analyses (PRISMA) flow diagram illustrating the study identification, screening, eligibility assessment, and inclusion process. *n* indicates the number of studies or references at each stage. PubMed, Cochrane, IEEE, and Google Scholar refer to the scientific databases used for study identification. Covidence refers to the software platform used to manage the study selection process and automatically detect duplicates. PRISMA refers to the Preferred Reporting Items for Systematic Reviews and Meta-Analyses guideline.

Google Scholar has limitations regarding the reproducibility of systematic searches, as it does not provide a fully standardized Boolean search interface or a complete export of all retrieved records. Therefore, the screening process was performed using the relevance-ranked results provided by the platform. This limitation was considered during the search process, and the search terms and eligibility criteria were predefined and consistently applied. The search strategy was aligned with the conceptual structure used in the other databases to maintain methodological consistency. Ultimately, 40 studies met the eligibility criteria and were included in the final review. Data extraction was performed using a predefined extraction form. No ongoing or awaiting classification studies were identified. The PRISMA flow diagram (Figure 1) summarizes the study selection process.

### 2.5 Architecture taxonomy

To provide a consistent framework for comparing the included studies, AI models were classified according to their dominant architectural design into seven operational categories. This taxonomy was developed to standardize terminology and facilitate comparisons across different artificial intelligence strategies.

- **Single-stage convolutional neural network (SCNN):** A single convolutional neural network performing the target task directly (e.g., classification or detection) without a distinct secondary module or task-specific sub-network (e.g., ResNet, DenseNet, VGG16, EfficientNet, and GoogleNet) (Sarvamangala and Kulkarni, 2021).
- **Detection-specific architecture (DET):** Deep learning models specifically designed for object detection or instance localization by generating bounding boxes or object proposals (e.g., YOLO family, RetinaNet, Faster R-CNN, SSD, and nnDetection) (Baumgartner et al., 2021; Tariq and Choi, 2025).
- **Encoder-decoder segmentation network (SEG):** Architectures performing pixel- or voxel-level segmentation using an encoder-decoder framework [e.g., U-Net and its variants, nnU-Net (Isensee et al., 2020), V-Net, DeepLab, and LinkNet].
- **Transformer-based architecture (TRANS):** Architectures in which self-attention is the principal mechanism for feature extraction and global context modeling [e.g., Vision Transformer (ViT), UNETR (Hatamizadeh et al., 2022), GCViT, and DINO-based models].
- **Multi-stage/hybrid pipeline (HYB):** Architectures combining two or more distinct computational modules within a unified diagnostic workflow. These include sequential pipelines (e.g., detection followed by segmentation or classification), multimodal fusion of imaging and non-imaging data (e.g., electronic health records), or combinations of deep learning and conventional machine learning components (Pang et al., 2024; Cao et al., 2024).
- **Ensemble architecture (ENS):** Approaches combining predictions from multiple independent deep learning models through voting, averaging, or score fusion without forming a sequential processing pipeline (e.g., combinations of YOLO, Faster R-CNN, SSD, RetinaNet, or multiple CNN backbones) (Alam et al., 2025).
- **Classical/non-deep-learning component (CLASSIC):** Studies relying wholly or partly on conventional algorithmic methods that are not trained end-to-end as deep neural networks (e.g., geodesic active contours and other model-based image analysis techniques) (Deo et al., 2024).

This taxonomy was applied retrospectively to all 40 included studies, as presented in Supplementary Table 2.

For studies evaluating multiple architectures, the classification was based on the primary clinical objective and the final intended output of the AI system rather than on the underlying architectural taxonomy. Accordingly, if the main clinical task was fracture classification, the study was categorized as *classification*; if the primary objective was fracture detection or localization, it was categorized as *detection*; and if the objective was fracture delineation or anatomical reconstruction, it was categorized as *segmentation*. Studies involving multiple clinical tasks (e.g., detection combined with classification or segmentation combined with classification), or those for which a single primary objective could not be clearly defined, were categorized as *hybrid*. Therefore, the term *hybrid architecture* in this review refers to a clinical task category rather than a specific neural network architecture.

### 2.6 Quality assessment

A comprehensive assessment of both methodological quality and clinical applicability was performed in order to evaluate the robustness and reliability of the synthesized evidence. The Quality Assessment of Diagnostic Accuracy Studies 2 (QUADAS-2) tool (Guni et al., 2024), widely used for evaluating risk of bias in diagnostic accuracy studies, was adopted as the foundational framework. The standard QUADAS-2 domains include:

1. patient selection,
2. index test,
3. reference standard, and
4. flow and timing.

Each domain was initially considered in terms of risk of bias and applicability concerns, particularly regarding population relevance, adequacy of the reference standard, and methodological transparency.

To better capture the specific characteristics of artificial intelligence-based diagnostic systems, the standard QUADAS-2 framework was retained in its original seven domain structure four risk-of-bias domains (patient selection, index test, reference standard, flow, and timing) and three applicability domains (patient, index test, and reference standard) but each domain was operationalized with AI-specific criteria rather than the generic QUADAS-2 signaling questions. Specifically, the *index test* domain incorporated assessment of training/validation split strategy, risk of data leakage, hyperparameter tuning transparency, and presence of external validation; the *flow and timing* domain incorporated assessment of artificial class rebalancing, data augmentation, and prevalence manipulation between training and test sets; the *reference standard* domain incorporated assessment of annotation protocol rigor (single vs. multiple annotators, consensus adjudication, inter-observer verification); and the three *applicability* domains incorporated assessment of dataset representativeness, PACS/DICOM workflow compatibility, and alignment of the AI output with the clinical decision the reference standard is meant to support. Each domain was scored on a four-point anchored scale (0–3), where 0 indicates low risk of bias/high applicability and 3 indicates unclear or unreported information (Supplementary Table 3).

All scoring criteria were predefined and operationalized using explicit and measurable indicators. Supplementary Table 3 presents the corresponding 0–3 scoring rubric, ensuring transparent and reproducible quality assessment across all included studies.

Quality assessment was performed independently by two reviewers. The assessment was conducted in a blinded manner to minimize subjective influence. Any discrepancies were resolved through discussion. Inter-rater reliability was evaluated using weighted Cohen's kappa, demonstrating substantial agreement (κ = 0.72).

Beyond classical diagnostic accuracy evaluation, this AI-specific extension incorporated additional dimensions relevant to modern deep learning systems. These included dataset quality (e.g., size, balance, and provenance), training and validation robustness (e.g., presence of external validation or patient-level splitting), performance metrics (e.g., AUC, accuracy, recall, and Intersection over Union), and clinical usability (e.g., interpretability and workflow integration). By combining traditional QUADAS-2 principles with AI-oriented evaluation criteria, this framework provides a more comprehensive and clinically meaningful assessment of AI-based fracture detection and segmentation studies.

## 3 Results

### 3.1 Study synthesis

**Figure 2:**
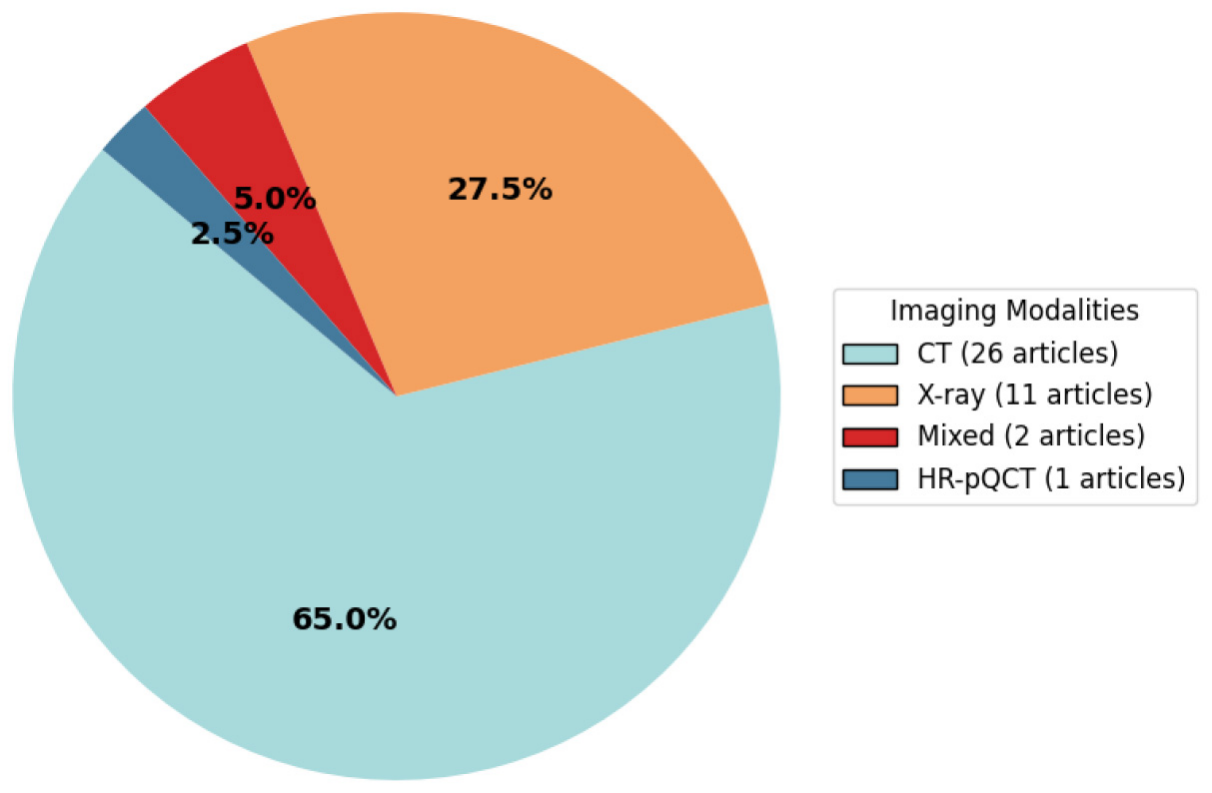
Distribution of imaging modalities across the included studies. The pie chart represents the proportion of included studies using each imaging modality for AI-based fracture analysis. Imaging modalities include computed tomography (CT), X-ray imaging, high-resolution peripheral quantitative computed tomography (HR-pQCT), and mixed-modality approaches. Percentages indicate the proportion of studies reporting each imaging modality.

A total of 40 studies were included in this review, focusing on the detection, classification and segmentation of complex and atypical fractures. These studies cover various types of fractures, including costal fractures, pelvic fractures, skull fractures, and unconventional vertebral fractures, detected and segmented using different imaging modalities, such as CT, and radiographs. To better understand the imaging modalities employed across the reviewed studies, Figure 2 presents their distribution, highlighting the predominance of CT and standard radiographs X-ray, while no study used MRI as the primary imaging modality.

This prevalence of CT and X-ray can be attributed to their respective advantages in clinical and technical contexts. Among the 40 studies reviewed, CT was the dominant imaging modality, used in 65% of the cases, compared to 27.5% relying on X-ray. Most studies rely primarily on CT (Wang, 2024; Nadeem et al., 2024; Gao et al., 2022) and standard X-rays (Turk et al., 2024; Oakden-Rayner et al., 2022). This preference is due to CT's high spatial resolution and 3D reconstruction capabilities, which allow for detailed visualization of complex bone structures, especially in challenging areas such as the pelvis, ribs, and spine (Zhou Q.-Q. et al., 2022). Numerous studies utilize three dimensional CT data acquired from trauma examinations to develop and train advanced AI models, particularly in applications such as pelvic fracture detection (Dreizin et al., 2021), Rib fracture segmentation (Jin et al., 2020) or the classification of vertebral fractures (Zakharov et al., 2023). At the same time, conventional radiography continues to be a predominant imaging modality in clinical settings, particularly for initial diagnostic assessment. The extensive availability of X-ray datasets enables the efficient development and validation of automated classification and segmentation algorithms, as evidenced by research in ankle fracture classification (Olczak et al., 2020). In contrast, studies using MRI (Oakden-Rayner et al., 2022) remained relatively scarce. MRI-based studies were less frequent among the included publications, whereas CT and conventional radiography were the most commonly used imaging modalities in fracture-related AI studies (Zhou Z. et al., 2022).

Consequently, AI models based on MRI images for the detection of complex fractures remain less developed to date.

In summary, the complementary strengths of CT, which provides high spatial resolution and 3D imaging, and the widespread availability and ease of use of standard radiographs explain the predominance of these two modalities in AI research focused on complex and atypical bone fractures. This trend is also supported by key studies that encompass a variety of anatomical regions and fracture types (Zhou Z. et al., 2022; Emon et al., 2022; Schilcher et al., 2024; Zdolsek et al., 2021).

To facilitate comparison across studies, Supplementary Table 4 presents a functional classification of the included studies according to their primary clinical objective and the corresponding clinical output delivered by each AI system. The table above is not a technical taxonomy based on model architecture (e.g., CNN, Vision Transformer, or U-Net) or imaging modality. Instead, it provides a functional classification of the included studies according to their main clinical objective. Studies are grouped by the diagnostic task they aim to automate, including detection, classification, segmentation, or a combination of these tasks. This approach focuses on the clinical output of each AI system rather than its technical design. For each study, the table highlights what the clinician receives, such as a diagnostic label (fracture presence or type), a bounding box showing the fracture location, a segmentation mask for quantitative analysis, or a structured report combining several outputs.

The Hybrid category is defined by clinical function rather than model architecture. It includes studies that integrate two or more clinically relevant tasks within a single workflow, such as fracture detection, severity classification, and anatomical localization (e.g., rib numbering), thereby providing a more comprehensive diagnostic output. For example, the pipeline proposed by Marting et al. (2025) was classified as Hybrid, whereas models performing only fracture detection were categorized as Detection.

This distinction reflects a growing trend toward integrated AI diagnostic workflows that better support clinical decision-making. However, combining multiple tasks also increases the complexity of validation, as errors may propagate across successive stages of the pipeline.

**Figure 3:**
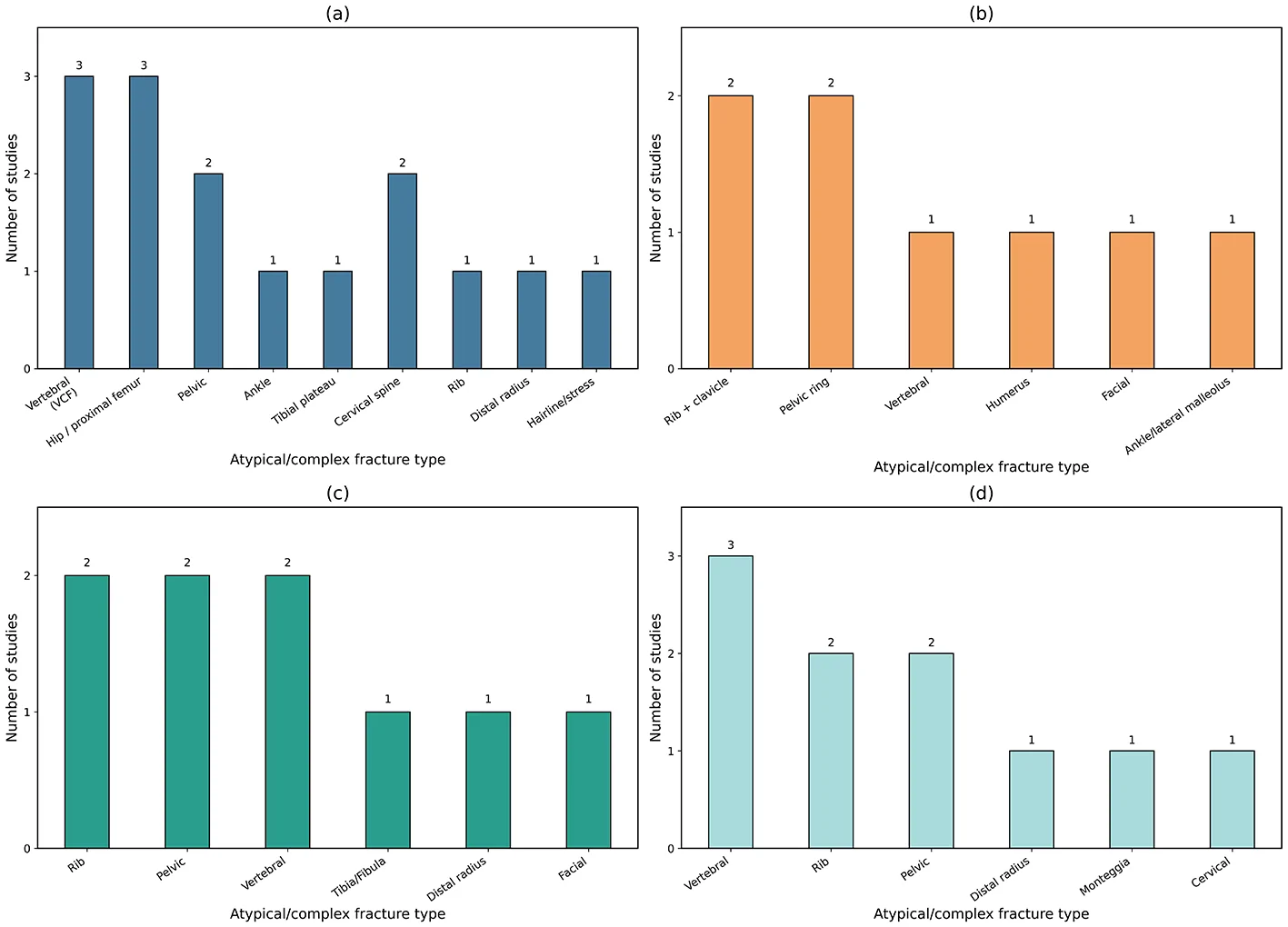
Distribution of reported fracture types and AI-based analysis tasks across the included studies. This bar chart summarizes 40 artificial intelligence (AI)-based studies classified according to their primary clinical objective. Studies are grouped into four functional categories: classification, detection, segmentation, and hybrid approaches. These categories are defined according to the clinical output provided by the AI system, illustrating the distribution of investigated tasks in fracture imaging. **(a)** Classification (*n* = 15), **(b)** Detection (*n* = 8), **(c)** Segmentation (*n* = 7), **(d)** Hybrid (*n* = 10).

To further clarify which fracture types are predominantly explored in the literature, Figure 3 presents the distribution of fracture types across 40 selected AI-based studies, grouped by task (classification, detection, segmentation, hybrid) and anatomical location or fracture complexity.

Classification-oriented studies mainly focus on atypical femoral fractures, particularly distinguishing Atypical Femoral Fractures (AFF) from Normal Femoral Fractures (NFF) (Schilcher et al., 2024; Zdolsek et al., 2021), as well as cranial (Emon et al., 2022), ankle (Olczak et al., 2020), pelvic (Dreizin et al., 2021), vertebral (Choi et al., 2023; Li et al., 2024), rib (Marting et al., 2025), pathologic proximal femur (Kim et al., 2021), distal radius (Oude Nijhuis et al., 2025), cervicothoracic junction (Yan et al., 2025), and hairline fractures (Jain et al., 2024). Detection-based studies predominantly target rib fractures (Zhou Q.-Q. et al., 2022; Zhou Z. et al., 2022; Cheng et al., 2024), pelvic fractures (Ukai et al., 2021; Rahman, 2024), Lateral Medial Anterior Fragment (LMAF) and Simple Fracture Only (SFO) groups (Liu J. et al., 2025), followed by vertebral (Zakharov et al., 2023), femoral (Oakden-Rayner et al., 2022), humeral (Verma et al., 2024), facial (Moon et al., 2024), cervical (Meadi et al., 2024; Nejad et al., 2023), and tibial plateau fractures (van der Gaast et al., 2025). Segmentation-focused research is mainly devoted to vertebral fractures (Rehman et al., 2019; Nadeem et al., 2024), while also including rib (Jin et al., 2020; Gao et al., 2022), tibial (Kim et al., 2023), radial (Ohs et al., 2021), thoracolumbar vertebral (Chen et al., 2024), pelvic (Nesheli et al., 2025), and complex or multi-type fractures (Turk et al., 2024). Finally, hybrid approaches combining multiple tasks have been applied to rib fractures (Zhou Q.-Q. et al., 2022), pelvic or multi-site fractures (Zeng et al., 2024; Dreizin et al., 2021), cervical fractures (Nejad et al., 2023), Monteggia fractures (Chakladar et al., 2026), and vertebral fractures (Chen et al., 2024; Glessgen et al., 2025). Overall, this distribution suggests that frequently encountered or anatomically complex fractures are generally investigated using task formulations that best reflect their clinical relevance and structural complexity.

### 3.2 Comparative study and model performances

Various AI models have been developed for fracture detection, classification, and segmentation. Supplementary Table 2 summarizes the main studies according to fracture type, model architecture, and reported performance metrics.

The results presented in Supplementary Table 2 indicate that AI performance varies considerably depending on the clinical task, fracture location, and model architecture. While some applications have reached a high level of maturity, others remain challenging.

For fracture detection, rib fractures show some of the best reported results, with sensitivities reaching 97.11% using RetinaNet-based hybrid approaches combining CNN features and object detection frameworks (Zhou Q.-Q. et al., 2022). Other rib fracture detection systems based on 3D segmentation networks and FROC evaluation have also demonstrated promising performance, with sensitivities ranging from 67.15% to 89.58% depending on the false-positive rate threshold (Jin et al., 2020; Zhou Z. et al., 2022). In contrast, pelvic fractures remain more difficult to identify, with reported AUC values between 0.74 and 0.89 despite the use of advanced hybrid frameworks combining Faster R-CNN and Bayesian causal models (Chen et al., 2025). These findings suggest that models optimized for one anatomical region may not generalize effectively to others.

A similar trend is observed in segmentation tasks. Vertebral fractures represent one of the most extensively studied categories and achieve excellent performance, with Dice coefficients reaching 0.984–0.997 using hybrid 3D U-Net-based frameworks for vertebral segmentation and fracture classification, including low-dose CT evaluation (Nadeem et al., 2024). Other vertebral segmentation approaches have reported Dice values above 0.92 and strong external validation performance, confirming the robustness of AI-assisted vertebral analysis (Rehman et al., 2019; Zakharov et al., 2023). Conversely, complex pelvic fractures exhibit lower performance, with reported sensitivity values around 0.730 for advanced 3D segmentation frameworks such as U-Net, ResNet-based networks, and UNETR architectures, reflecting the difficulty associated with irregular and fragmented fracture patterns (Nesheli et al., 2025).

Hybrid frameworks combining detection, segmentation, and classification tended to report more balanced results across the reviewed studies. For example, integrated approaches using FDD-Net with deformable multi-scale fusion achieved Dice scores between 0.935 and 0.955 for pelvic fracture segmentation and classification (Zeng et al., 2024), while combined segmentation-classification models for thoracolumbar vertebral fractures achieved segmentation DSC values around 0.90 and classification AUC values up to 0.96 (Chen et al., 2024). However, direct head-to-head comparisons between architectures remain rare, and comparisons across studies must be interpreted with caution because of differences in datasets, annotation protocols, validation strategies, and reported evaluation metrics.

Several deep learning architectures have demonstrated strong performance in specific clinical scenarios. FracNet, based on 3D U-Net architecture, achieved high sensitivity for rib fracture detection and segmentation (Jin et al., 2020). Similarly, FDD-Net with DMF demonstrated competitive results for complex pelvic fracture analysis (Zeng et al., 2024). For proximal femoral fractures, DenseNet-based and transformer-based models achieved very high discrimination performance, with external validation AUC values reaching 0.98–0.99 and performance comparable to radiologists (Oakden-Rayner et al., 2022; Kim et al., 2021). Transformer-based architectures and hybrid vision models, including UNETR and GCViT-based frameworks, also achieved competitive results for fracture segmentation and cervical spine fracture detection, although they generally require larger annotated datasets and higher computational resources (Nesheli et al., 2025; Nejad et al., 2023).

Overall, the reviewed studies demonstrate that no single architecture consistently outperforms others across all fracture types and imaging modalities. Instead, performance remains strongly dependent on the anatomical target, imaging modality, clinical task, and validation strategy. These observations highlight the importance of task-specific model selection and the urgent need for standardized evaluation protocols to enable reliable comparison and translation of AI systems into clinical workflows.

### 3.3 Cross-dimensional synthesis

To move beyond a purely descriptive summary, findings were further compared across four dimensions: clinical task (classification, detection, segmentation, or hybrid), imaging modality, fracture type, and validation level (internal, external, or prospective). This comparison shows that model performance depends not only on the deep learning architecture but also on these factors. Classification models with high performance (AUC ≥ 0.9) were mainly reported for simple fracture classification tasks using a single imaging modality, such as X-ray images of cranial, ankle, and cervical fractures, with internal validation. In contrast, studies using external or prospective validation (Schilcher et al., 2024; Oakden-Rayner et al., 2022) showed a small decrease in performance, although the results remained clinically useful. Detection models on CT images, especially for rib and pelvic fractures, generally achieved higher sensitivity than segmentation models. This difference is explained by the greater difficulty of accurately outlining complex and multi-fragment fractures.

Clinical readiness was defined based on the validation strategy, dataset heterogeneity, and clinical applicability, ranging from early-stage experimental models (internal validation only) to clinically translatable models (external or prospective validation using independent and heterogeneous datasets). Because no standardized framework currently exists to classify the clinical maturity of deep learning studies for fracture diagnosis, we developed a pragmatic readiness classification specifically for this systematic review. This classification was informed by methodological principles recommended in established AI reporting and evaluation guidelines, including CLAIM (Mongan et al., 2020), TRIPOD+AI (Collins et al., 2024), and DECIDE-AI (Vasey et al., 2022), which emphasize the importance of validation design, generalizability, and clinical evaluation. Accordingly, the proposed readiness tiers reflect increasing levels of confidence in the robustness and potential clinical translation of the models rather than regulatory approval or routine clinical implementation. Specifically, the readiness tiers were defined as follows:

- Tier 1 (Technical feasibility): internal validation only, with single- or multi-center training datasets and no independent external test set.
- Tier 2 (Reader-benchmarked/Pre-clinical): internal validation supplemented by expert or reader comparison, multi-center internal datasets, or limited external validation.
- Tier 3 (Externally/Prospectively validated): validation on an independent external cohort or prospective dataset.

No included study reported regulatory clearance; tier labels reflect validation design only, not regulatory or deployment status. The clinical readiness classification of all included studies is presented in Supplementary Table 5.

Among the 40 included studies, 18(45%) were classified as Tier 1, reflecting reliance on internal validation only; 11(27.5%) were classified as Tier 2, incorporating reader comparison or partial external testing without a fully independent cohort; and 11(27.5%) were classified as Tier 3, reporting validation on an external or prospective cohort. Tier 3 classifications were concentrated in a limited number of anatomical regions and tasks, notably rib fracture detection (Zhou Q.-Q. et al., 2022; Marting et al., 2025), atypical femoral fracture classification (Schilcher et al., 2024), proximal femoral fracture detection (Oakden-Rayner et al., 2022), and selected pelvic and vertebral applications (Zeng et al., 2024; Chen et al., 2024; Kim et al., 2021; Liu J. et al., 2025). Tier 2 studies were more evenly distributed across tasks and modalities, suggesting an intermediate stage of validation that is common but insufficient on its own to support clinical deployment. Notably, no study across any tier reported regulatory clearance, underscoring that even Tier 3 classifications reflect a research-grade level of external validation rather than clinical or regulatory readiness. This distribution reinforces the conclusion that, despite encouraging performance metrics reported in several studies, a substantial share of the evidence base for atypical and complex fracture diagnosis remains at an early, single-center stage of validation.

### 3.4 Methodological quality review

**Figure 4:**
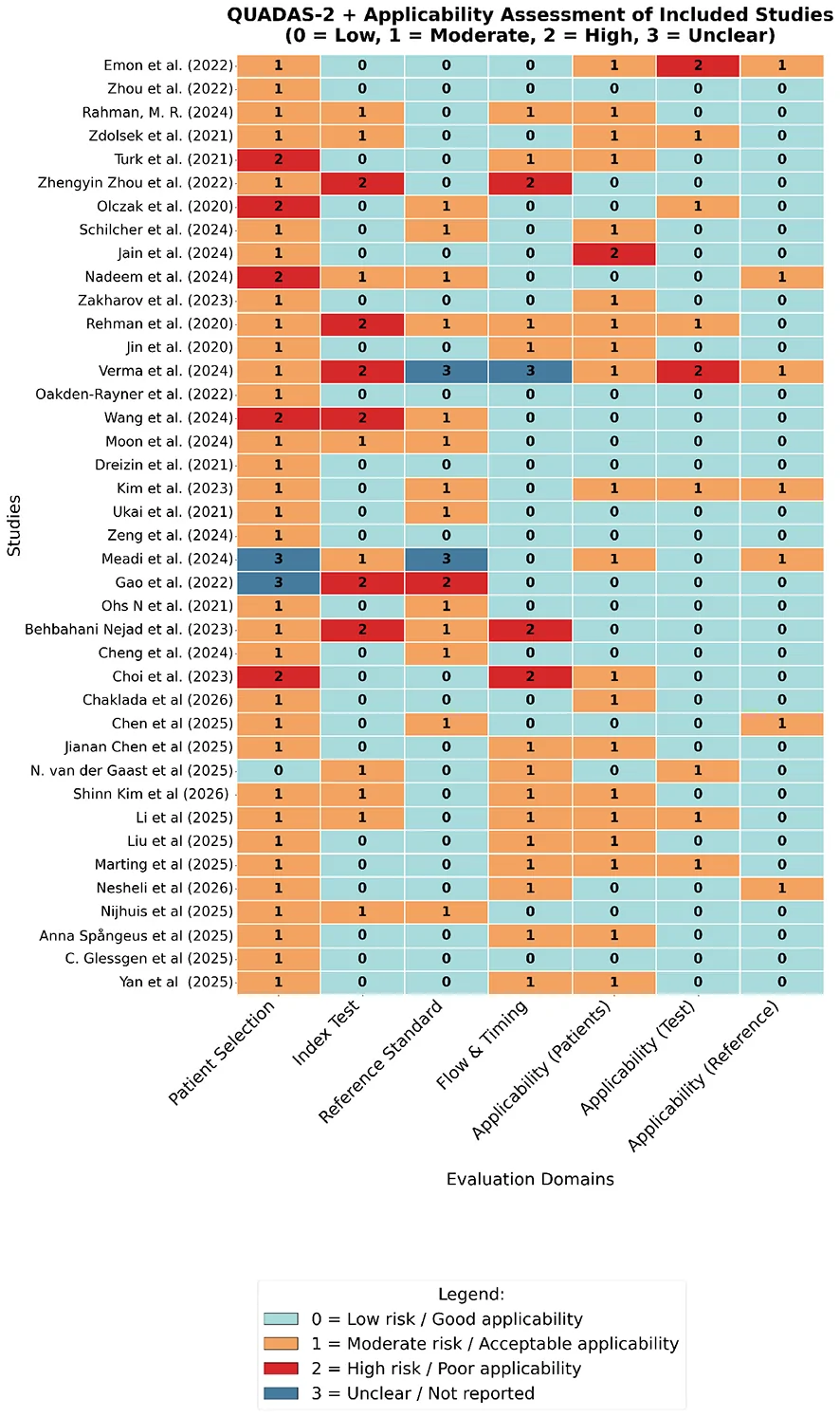
Quality and applicability assessment using the Quality Assessment of Diagnostic Accuracy Studies 2 (QUADAS-2) tool.This heatmap presents the QUADAS-2 risk-of-bias and applicability assessments for each included study. Scores from 0 to 3 represent increasing levels of risk of bias or applicability concerns. Blue indicates low risk of bias or low applicability concerns, orange indicates moderate concerns, red indicates high risk of bias or high applicability concerns, and dark blue indicates unclear or unreported information. The visualization summarizes methodological strengths and limitations across the included studies.

To provide a comprehensive overview of the results, Figure 4 presents a detailed summary of the risks of bias and applicability concerns assessed using the QUADAS-2 tool in all included studies. This visualization highlights key methodological strengths and limitations identified in the analysis. Note: This figure presents quality and applicability scores (0–3) for each study, where blue indicates low risk/high applicability, orange moderate risk, red high risk/low applicability, and dark blue unclear or unreported data.

An analysis of 40 studies using the QUADAS-2 framework reveals common sources of bias, primarily related to patient selection and study design. Several studies applied strict exclusion criteria, such as omitting patients with implants, pathological fractures, pediatric cases, or repeated examinations (Wang, 2024; Olczak et al., 2020), which may limit the representativeness of the evaluated populations. Additional methodological concerns included small datasets, class imbalance, and annotation variability, which may affect model performance, particularly for rare or atypical fractures (Turk et al., 2024). Even high-performing AI models face challenges. Some relied on two-dimensional (2D) imaging alone or lacked integration with other modalities, affecting accuracy in complex cases (Verma et al., 2024). Few studies systematically compared AI with traditional machine learning, and performance often declined on independent external datasets, demonstrating operating point shifts (Wang, 2024; Oakden-Rayner et al., 2022). Reference standards were sometimes unreliable due to inter-expert variability or lack of surgical confirmation (Emon et al., 2022; Zhou Q.-Q. et al., 2022), and single-annotator datasets or aggressive augmentation could introduce bias (Gao et al., 2022; Kebaili et al., 2023). Despite these limitations, some studies demonstrated strong methodological rigor. For example (Schilcher et al., 2024) included 1,073 patients from 72 hospitals with repeated cross-validation, although the predominantly Caucasian cohort limits broader applicability. Most studies maintained clear training/testing splits (Jin et al., 2020), but homogeneous patient groups restricted generalizability (Schilcher et al., 2024; Jain et al., 2024; Nadeem et al., 2024). Promising strategies include 3D segmentation aligned with clinical workflows (Ukai et al., 2021) and multimodal fusion with electronic health records (Schilcher et al., 2024). Overall, these findings highlight AI's strong potential for fracture detection and segmentation, while emphasizing the need for prospective studies, standardized methodology, and diverse datasets to ensure reliable clinical translation. An additional critical aspect of data quality concerns the annotation process, which directly influences the reliability of the ground truth used for model development and evaluation. Data quality analysis reveals substantial variability in annotation protocols across studies. While some investigations rely on consensus annotations from multiple expert radiologists (Zakharov et al., 2023; Dreizin et al., 2021), others provide limited information regarding annotator expertise or use annotations with insufficient clinical validation (Turk et al., 2024; Meadi et al., 2024). This heterogeneity, particularly the frequent absence of double reading or expert consensus, may affect the reliability of the ground truth and limit the clinical generalizability of AI models.

## 4 Discussion

### 4.1 Results interpretation

#### 4.1.1 Quantitative synthesis of validity threats

To complement the study-level descriptive synthesis, we systematically assessed the proportion of included studies that met minimal EBM standards for diagnostic AI validation. Despite the frequent use of internal validation approaches, external validation remains one of the major challenges in this field. External validation, defined as the evaluation of a model on data obtained from institutions that were not involved in model development or training, provides a stronger assessment of generalizability and better reflects potential performance in real-world clinical settings.

Among the 40 included studies, 11(27.5%) reported evaluation on an externally sourced validation dataset, while only 1 study (2.5%; Chen et al., 2024) incorporated prospective validation. The remaining 29 studies (72.5%) did not report external validation and relied mainly on internal validation strategies, including retrospective datasets with held-out test sets or cross-validation procedures derived from the same underlying cohorts. Although these approaches are useful for preliminary model development and performance estimation, they may provide limited evidence regarding clinical generalizability, as they do not fully account for variations in patient populations, imaging protocols, scanner characteristics, and clinical workflows encountered during real-world deployment. These findings are summarized in Supplementary Tables 5, 6.

More specifically, among the 40 included studies, monocentric designs remained the most common. A total of 16 studies (40%) were conducted in a single institution, while 9 studies (22.5%) involved two centers. In contrast, 12 studies (30%) included more than two centers and were considered multicentric, while the number of participating centers was not clearly reported in 3 studies (7.5%). Although multicenter studies provide greater variability in terms of scanners, acquisition protocols, and patient characteristics, the presence of multiple centers alone does not necessarily guarantee independent validation, as discussed below.

Regarding study design, the evidence base was largely dominated by retrospective investigations. Indeed, 39 out of the 40 included studies were retrospective, with only one exception (Chen et al., 2024), which used a mixed design combining retrospective model development with prospective validation. This prospective component provides stronger evidence for clinical applicability, as it allows evaluation of model performance in a more realistic setting and may reduce some of the selection and annotation biases commonly associated with retrospective datasets. Nevertheless, internal validation approaches, such as train/validation/test splits or k-fold cross-validation, remain widely used and represent the current standard methodological practice in AI model development.

Moreover, it is important to distinguish between multicenter design and true external validation. Several multicenter studies (Nadeem et al., 2024; Nejad et al., 2023; Schilcher et al., 2024) used datasets collected from multiple institutions within the same registry or collaborative network, where contributing centers participated in model development. While such approaches improve data diversity and reduce dependence on a single institution, they do not represent fully independent external validation. This distinction is essential when interpreting the level of evidence provided by multicenter studies, as true generalizability requires evaluation on independent populations that reflect the variability encountered in real-world clinical practice.

Independent held-out test sets were reported in the majority of studies, reflecting generally appropriate evaluation practices. However, a limited number of studies relied exclusively on cross-validation without a separately defined independent test cohort (Chen et al., 2025; Ohs et al., 2021; Zdolsek et al., 2021). Although cross-validation is an accepted evaluation strategy, the absence of an independent test set may lead to optimistic estimates of model performance because repeated resampling within the same patient population can increase the risk of subtle information leakage, particularly when strict patient-level separation is not ensured.

Direct comparison with clinicians through reader studies, arguably the most clinically relevant benchmark, was reported inconsistently across the literature. The most rigorous studies compared AI performance with experienced radiologists or orthopedic surgeons using formal reader-study designs and, in several cases, demonstrated performance comparable to or exceeding that of expert clinicians (Zhou Q.-Q. et al., 2022; Oakden-Rayner et al., 2022; Kim et al., 2021; Liu J. et al., 2025). In contrast, many technically oriented studies evaluated models primarily against competing AI approaches rather than human experts (Gao et al., 2022; Zeng et al., 2024; Nejad et al., 2023; Meadi et al., 2024; Nesheli et al., 2025). Although algorithm-to-algorithm comparisons provide insight into technical advancement, they offer limited evidence regarding clinical utility, as improvement over another computational model does not necessarily translate into improved patient care or workflow integration.

Overall, these findings indicate that AI research in fracture detection has reached a relatively mature level of internal methodological evaluation, as demonstrated by the widespread adoption of internal validation procedures and independent test sets. Nevertheless, two critical requirements for clinical translation remain insufficiently addressed: robust external validation using geographically and temporally independent cohorts, and rigorous head-to-head comparisons with clinicians. Only a limited subset of studies simultaneously incorporated the four strongest methodological characteristics: multicenter data collection, true external validation, an independent held-out test set, and clinician comparison (Zhou Q.-Q. et al., 2022; Oakden-Rayner et al., 2022; Oude Nijhuis et al., 2025; Cheng et al., 2024; Liu J. et al., 2025; Kim et al., 2021; Chen et al., 2024). These studies currently represent important methodological benchmarks for future research aiming to develop clinically robust and generalizable AI systems for fracture detection.

#### 4.1.2 Imaging modalities

Among the 40 studies analyzed, CT is the most frequently used imaging modality, followed by X-ray (Figure 2). No study focused exclusively on MRI. This predominance of CT is explained by its ability to produce high-resolution thin-slice images, enabling precise three-dimensional reconstruction of bone structures and improved visualization of complex or subtle fractures (Gao et al., 2022; Zdolsek et al., 2021). Conversely, despite MRI's superior sensitivity for detecting bone marrow edema and soft-tissue injuries, its underrepresentation may be attributed to longer acquisition times, complex multiparametric sequences, and limited integration into emergency protocols (Zhou Z. et al., 2022). The limited number of MRI-based studies may also be explained by its predominant role in soft-tissue assessment, higher cost, lower availability, and longer acquisition times in emergency settings (Zhou Z. et al., 2022). This absence of studies centered on MRI highlights a significant research gap, especially with regard to the detection of invisible subtle fractures on CT. In Schilcher et al. (2024), standard X-ray images were used, combined with clinical data from electronic health records, demonstrating the value of multimodal data fusion.

#### 4.1.3 Algorithmic performance

Algorithmic performance in fracture analysis is highly dependent on fracture type, anatomical complexity, AI architecture, task definition, and evaluation strategy. As presented in **Table 5**, for classification tasks, 2D convolutional neural networks, including ResNet, VGG, and ResNeXt, consistently demonstrate high performance for visually distinct fractures such as cranial, cervical, and ankle fractures, often achieving AUC values ≥0.9 and occasionally surpassing radiologist performance (Emon et al., 2022; Olczak et al., 2020; Meadi et al., 2024).

Multimodal deep learning approaches, which integrate imaging with clinical features, have been reported to enhance sensitivity and AUC for subtle or rare fractures, such as AFF versus NFF, in at least one cohort. For instance, a ResNet50-based multimodal fusion model improved sensitivity from 79.6% to 90.3% and AUC from 0.966 to 0.987 (Schilcher et al., 2024; Zdolsek et al., 2021); however, as discussed below, this finding currently rests on a single research group's data and should be interpreted as preliminary. EfficientNet, MobileNet, and MnasNet have also shown strong classification performance for fresh versus non-fresh vertebral fractures, outperforming junior spine surgeons and approaching expert-level accuracy (Li et al., 2024). Similarly, models like YOLOv12 demonstrated exceptional detection and classification capabilities for lateral malleolar avulsion fractures and subfibular ossicles on CT scans, achieving high mAP50 and AUC values, surpassing experienced radiologists (Liu J. et al., 2025). These results illustrate that CNN-based architectures remain highly effective for well-defined fractures and tasks emphasizing rapid classification. It should be noted, however, that these comparisons are drawn from heterogeneous, largely single-center studies using non-standardized metrics, and should not be interpreted as direct evidence of one architecture's superiority over another. Conversely, transformer-based classification of vertebral compression fractures using weakly supervised approaches (e.g., MULAN) achieved comparatively modest sensitivity (0.770) despite high specificity (0.981) (Choi et al., 2023), illustrating that even within the vertebral/spinal region, high performance is not guaranteed across architectures or tasks.

Detection-oriented models, including YOLO, RetinaNet, DenseNet, and nnDetection, have proven efficient for anatomically repetitive fractures, such as ribs, femur, humerus, and nasal bones, achieving high sensitivity and reducing clinical reading time (Zhou Q.-Q. et al., 2022; Oakden-Rayner et al., 2022; Verma et al., 2024; Wang, 2024; Marting et al., 2025). Nonetheless, their performance declines in low-contrast or anatomically complex regions, such as facial or pelvic fractures, where fast inference can compromise accuracy (Moon et al., 2024; Dreizin et al., 2021). Similarly, GoogleNet and ResNet models reliably detect tibial plateau fractures but struggle with subtype classification according to Schatzker grading, highlighting the challenge of fracture heterogeneity and uneven data distribution (van der Gaast et al., 2025).

Segmentation models leveraging 3D architectures, such as 3D U-Net, FU-Net, DeepLab, V-Net, and nnU-Net, deliver excellent results in fracture quantification and treatment planning. These approaches consistently produce high Dice scores (often >0.95), robust reproducibility under low-dose imaging, and precise anatomical delineation for vertebral, tibial, radial, and pelvic fractures (Rehman et al., 2019; Nadeem et al., 2024; Kim et al., 2023; Ohs et al., 2021; Ukai et al., 2021; Nesheli et al., 2025). Advanced segmentation algorithms, including multi-orientation 2D reconstruction and 3D-GAC, enhance detection in complex anatomical regions, while transformer-based models (UNETR) demonstrate high specificity and rapid inference, though sometimes at the cost of sensitivity in fragmented areas (Ukai et al., 2021; Moon et al., 2024; Nesheli et al., 2025). Notably, segmentation-focused models such as a scaphoid fracture detection model effectively identify subtle or occult fractures, detecting 41% of cases missed by experts, although they still generate false positives (Chakladar et al., 2026).

Hybrid models that integrate detection, segmentation, and classification do not perform uniformly across fracture types, and this heterogeneity should temper any general claim of superiority. For pelvic fractures, hybrid dual-stream segmentation frameworks (e.g., FDD-Net+DMF) achieved high Dice scores (0.935–0.955) (Zeng et al., 2024), but a separate hybrid pipeline for pelvic detection and Tile AO/OTA grading using Faster R-CNN combined with a Bayesian causal model reached only moderate discrimination (AUC 0.74–0.89) in a cohort of 373 scans (Chen et al., 2025). For vertebral fractures, hybrid nnU-Net+ResNet18 pipelines achieved high sensitivity (90.4%) but comparatively lower specificity (80.5%) (Glessgen et al., 2025), indicating a trade-off rather than uniformly “excellent” performance. For cervical spine fractures, hybrid GCViT+YOLOv8 pipelines reported high accuracy (Macro F1 0.96) (Nejad et al., 2023), although further evaluation across diverse datasets is needed to confirm the robustness of these findings. Taken together, hybrid architectures appear best suited to tasks combining morphological localization with clinical grading, but their reported performance advantage is anatomy- and task-specific rather than general, and in several cases (e.g., pelvic detection) hybrid pipelines did not outperform simpler single-stage architectures reported elsewhere in Supplementary Table 2. Similarly, the finding that multimodal fusion of imaging and EHR data improves sensitivity and AUC for atypical femoral fracture classification is derived from a single research group and overlapping study cohorts (Schilcher et al., 2024; Zdolsek et al., 2021). While the reported improvement (sensitivity from 79.6% to 90.3%; AUC from 0.966 to 0.987) is notable and is supported by a large dataset (1,073 patients across 72 hospitals), it should be regarded as preliminary, hypothesis-generating evidence for the potential value of multimodal fusion rather than a definitive generalizable finding, given that independent confirmation across different populations or fracture types was not identified among the 40 included studies.

Despite these advancements, challenges remain. Biomechanically complex fractures, such as pelvic or comminuted fractures, still exhibit reduced sensitivity, and models often rely on labor-intensive manual annotations, which introduce inter-observer variability and bias (Zakharov et al., 2023; Zhou Q.-Q. et al., 2022; Rehman et al., 2019). Smaller datasets and limited cross-dataset validation further constrain generalizability, especially for segmentation-only or computationally heavy hybrid architectures. Approaches like MULAN, which leverage image-level labels and relevance-CAM techniques, demonstrate a practical pathway to robust, annotation-efficient vertebral fracture detection (Rehman et al., 2019). Other studies, such as MSDA-MLNet for cervicothoracic junction fractures, highlight the benefit of multi-view image fusion, edge enhancement, and transfer learning to improve accuracy and reduce training time (Yan et al., 2025).

Overall, deep learning in fracture analysis shows considerable promise in augmenting clinical decision-making, enhancing diagnostic accuracy, and accelerating assessment workflows. Classification networks excel in visually distinct and repetitive fractures, and segmentation models provide precise volumetric delineation for treatment planning; hybrid architectures can bridge detection, anatomical detail, and clinical interpretability, but, as shown above, this advantage is task and anatomy specific rather than general, and should not be assumed to extend uniformly across fracture types. Nevertheless, the persistent challenges of complex fracture morphology, heterogeneous datasets, and annotation dependency underscore the need for continued methodological innovation to improve robustness, generalization, and practical clinical deployment.

#### 4.1.4 Limitations and methodological concerns

A common methodological limitation across many studies is the exclusion of occult fractures, with most analyses restricted to radiographically visible cases. Patients with implants, pediatric fractures, repeated examinations, or pathological fractures were frequently omitted, introducing selection bias that likely inflates reported model performance and limits generalizability in real-world settings (Zhou Q.-Q. et al., 2022; Olczak et al., 2020; Turk et al., 2024). Metric definitions and reporting also vary considerably: detection and classification studies typically report AUC or sensitivity, whereas segmentation studies rely on Dice or IoU scores, complicating direct comparisons. Furthermore, most studies depend on small, single-center datasets with limited external validation, further restricting reliability and applicability (Olczak et al., 2020; Wang, 2024; Zhou Q.-Q. et al., 2022). One study highlighted the challenges of small sample sizes, annotation bias, and limited fracture-type detail, while still demonstrating AI potential in nasal fracture assessment (Wang, 2024). Few studies reported subgroup analyses (e.g., osteoporotic versus non-osteoporotic, pediatric vs. adult), limiting understanding of model performance across diverse patient populations (Chakladar et al., 2026; Chen et al., 2025).

Segmentation remains particularly challenging because of fracture complexity. One study noted limitations arising from the small, heterogeneous FracAtlas dataset, inconsistent annotations, and the need to crop images to optimize performance (Turk et al., 2024). Even advanced models, such as DeepLab v3+ with ASPP and ResNet-50, occasionally merge or misclassify fragments, introducing minor masking errors that affect 3D reconstruction quality (Kim et al., 2023). These models are highly dependent on data quality, primarily trained on tibia and fibula datasets, and require substantial GPU resources, raising questions regarding generalization to other bones. Despite these limitations, automatic segmentation offers faster, reproducible, and more reliable 3D visualization, supporting preoperative planning and fracture analysis. However, reproducibility was not fully assessed for fractured bones in some studies (Ohs et al., 2021), and very low-quality images remain difficult to segment. Additionally, certain models focus only on specific bone regions (e.g., proximal radius) and do not perform fracture localization, potentially missing subtle features. External validation is often lacking, and most datasets include limited ethnic diversity, emphasizing the need for broader testing (Schilcher et al., 2024).

Several studies exhibit specific methodological weaknesses that affect reliability and generalizability. One rib-fracture detection study using CFSG U-Net suffered from incomplete validation, producing a high number of false positives, limited training size, and absence of external validation, with data sourced from a single center (Zhou Z. et al., 2022). Another study relied on a conventional rib preprocessing and Region Extraction (RE) module, introducing fragility as errors propagated through the pipeline, especially for elongated or thin fractures (Gao et al., 2022). Similarly, one cranial fracture study used a validation approach splitting data by image rather than patient, leading to potential data leakage and artificially inflated performance; the study also faced small, imbalanced datasets, high computational demands, and information loss during preprocessing, resulting in high false-positive rates requiring human verification (Emon et al., 2022; Jin et al., 2020). Another vertebral fracture study trained the model on data from a single hospital and focused exclusively on the lumbar spine, with annotations provided by a single radiologist, raising concerns regarding generalizability and inter-reader variability (Choi et al., 2023).

Studies focusing on pelvic and vertebral fractures similarly face dataset and methodological constraints. One interpretable model for Tile AO/OTA pelvic fracture grading was developed using only 373 CT scans from two level-I trauma centers, with manual cropping and preselected fracture types potentially introducing bias (Chen et al., 2025). Factors such as osteoporosis or prior pelvic surgery were not considered, and high-confidence thresholds risk missing subtle or non-displaced fractures. Other studies also highlighted limitations including single-center datasets, exclusion of complex or pathologic cases, reliance on manual Region of Interest (ROI) selection, and lack of incorporation of clinical features, all affecting generalizability (Glessgen et al., 2025; Chen et al., 2024). Additional work on pathologic proximal femur and vertebral compression fractures similarly faced issues with single imaging modalities, manual cropping, and limited interpretability, emphasizing that these tools support rather than replace clinical judgment (Kim et al., 2021; Li et al., 2024).

Other studies illustrate challenges in model generalizability and technical constraints. Research on LMAF vs. SFO and automated rib fracture analysis encountered small or imbalanced datasets, exclusion of complex cases, and modality-specific limitations (CT only), affecting performance across diverse populations (Liu J. et al., 2025; Marting et al., 2025). Another study demonstrated that transformer-based models require large datasets for optimal performance and face computational demands that limit real-world deployment (Nesheli et al., 2025). Similarly, studies on distal radius fractures, geriatric CT imaging, Schatzker classification, and multi-scale lumbar networks all reported constraints related to dataset size, single-center data, annotation quality, modality dependence, and lack of external validation, highlighting the overarching need for multicenter, diverse, and rigorously validated datasets (Oude Nijhuis et al., 2025; Spångeus et al., 2025; van der Gaast et al., 2025; Yan et al., 2025).

In summary, while AI models show promising performance in fracture detection, classification, and segmentation, common limitations include small and homogeneous datasets, single-center data, manual preprocessing, modality-specific constraints, incomplete validation, annotation bias, and limited external testing. These challenges collectively restrict generalizability, clinical applicability, and reproducibility, emphasizing the need for large, diverse, and rigorously validated datasets to ensure reliable deployment in real-world clinical settings.

### 4.2 Challenges and opportunities for clinical implementation

The findings highlight the strong potential of AI, particularly advanced architectures, to enhance the detection, classification, and segmentation of complex and atypical fractures (Rahman, 2024). Despite this promise, clinical adoption remains limited due to several challenges. Selection bias is a major concern, as many studies rely on single-center datasets with uniform imaging protocols and often exclude atypical or difficult cases, restricting generalizability to real-world trauma and geriatric populations (Turk et al., 2024; Olczak et al., 2020; Wang, 2024). Ethical and legal uncertainties, including liability for missed diagnoses and management of false positives, further complicate implementation. Future research should prioritize prospective, multicenter trials, integration of relevant clinical variables (e.g., age, medical history, injury type), and the development of standardized datasets, transparent benchmarks, and interpretable models tailored to real-world practice.

Several studies demonstrate the technical potential of AI pipelines for fracture assessment. Nejad et al. (2023) proposed a holistic pipeline combining vertebrae classification and fracture localization using the GCViT, which captures long-range dependencies through global attention and achieves high accuracy (MacroF1: 0.96) compared with conventional CNNs. However, its quadratic computational complexity and 14.7 million non-trainable parameters impose substantial memory and data requirements, limiting deployment in resource-constrained clinical settings. Similarly, models extending 2D approaches to 3D fracture detection (Ukai et al., 2021) can reduce radiologists' workload but are hindered by long processing times, sensitivity to metallic artifacts, reliance on manual annotations, and the need for high-performance GPUs. Despite these limitations, AI demonstrates clear opportunities for improving diagnostic assistance, accelerating detection, and enabling early intervention, particularly when combined with segmentation models (Schilcher et al., 2024; Zhou Q.-Q. et al., 2022; Jin et al., 2020).

Interpretable AI models play a crucial role in fostering clinician trust and enabling human–machine collaboration. Automation of pelvic fracture severity scoring (Chen et al., 2025, 2024) enables rapid triage of polytrauma patients, but clinical adoption requires seamless integration with existing workflows, validation across diverse populations, and careful management of false negatives, especially given anatomical variability in elderly patients. Nadeem et al. (2024) demonstrated accurate detection of vertebral compression fractures using Genant's morphometric criteria, yet computation time (7 minutes per scan) and population specificity (older COPD patients) limit generalizability. MULAN (Choi et al., 2023), segmentation-based deep learning models (Chakladar et al., 2026), and transformer-based pelvic fracture segmentation (Nesheli et al., 2025) similarly face challenges including multi-hospital validation, imaging variability, and processing efficiency, while offering opportunities for workload reduction, improved early detection, and enhanced interpretability.

The performance of deep learning architectures varies depending on task and context. EfficientNet, MobileNet, and MnasNet face difficulties with variable X-ray quality, vertebral morphology, and soft tissue artifacts, while manual ROI cropping and lack of automatic localization limit workflow integration (Li et al., 2024). YOLOv12 shows high accuracy for LMAF and SFO on CT scans (Liu J. et al., 2025), but misclassifications due to anatomical variants and limited training data reduce generalizability. Marting et al. (2025) highlighted challenges in classifying underrepresented fracture types and processing time constraints, yet opportunities exist in standardized classification and enhanced communication among clinicians. Similarly, distal radius fracture classification (AO/OTA) (Oude Nijhuis et al., 2025) faces moderate accuracy for complex fractures and depends on high-quality radiographs, but can assist rapid identification of extra-articular fractures and improve workflow consistency.

AI algorithms designed for hospital integration, such as IB Lab FLAMINGO (Spångeus et al., 2025) and MSDA-MLNet (Yan et al., 2025), demonstrate both challenges and opportunities. Implementation requires careful workflow integration, radiologist training, and quality assurance to control false positives. Differences in imaging protocols, scanner types, and patient profiles necessitate robust calibration and validation, but the potential benefits include improved detection of underreported fractures, more efficient referral pathways, faster grading, and support for emergency and trauma care resource allocation. Across these applications, AI is best positioned as an intelligent support tool, augmenting rather than replacing clinicians, providing rapid visualization, risk stratification, and real-time decision support.

Overall, AI demonstrates substantial promise in fracture detection, classification, and segmentation, particularly when combining advanced architectures with interpretable outputs. Challenges remain in dataset variability, computational requirements, real-world validation, and integration into clinical workflows. Nevertheless, the opportunities for enhancing patient care, reducing diagnostic delays, supporting non-expert clinicians, and improving decision-making are significant, highlighting the importance of thoughtful development, prospective evaluation, and seamless clinical integration.

Furthermore, recent efforts toward multicenter dataset harmonization provide a promising direction for overcoming the persistent limitations of single-center databases. Integrating multiple public datasets into a unified upper-limb fracture resource has demonstrated the feasibility of increasing anatomical diversity, patient heterogeneity, and representation of clinically complex fracture patterns (Atitallah et al., 2026). Such initiatives can improve model robustness and external validity while reducing bias associated with homogeneous populations and institution-specific imaging protocols. However, rare and highly complex fracture cases remain underrepresented because of the continuing scarcity of publicly available annotated datasets. Expanding collaborative multicenter repositories with standardized annotations and broader demographic coverage will therefore be essential for translating AI systems into reliable real-world clinical practice.

### 4.3 Implementation science considerations for AI-based fracture detection

Accuracy of AI models for fracture detection, successful translation into routine clinical practice depends on much more than algorithmic performance. The implementation of AI systems in healthcare is influenced by technical, organizational, regulatory, and human factors that extend beyond model development. Guided by the NASSS framework (Greenhalgh et al., 2017), we identify four major implementation challenges that emerge for AI applications targeting atypical and complex fractures.

- **Technology integration and clinical workflow**.
  Most studies included in this review developed and evaluated AI models as standalone research tools rather than as components of integrated clinical imaging ecosystems. Only a limited number of studies reported inference times or outputs compatible with Picture Archiving and Communication Systems (PACS) or Radiology Information Systems (RIS), thereby limiting their immediate clinical applicability (Zhou Q.-Q. et al., 2022; Ukai et al., 2021). In real-world practice, AI systems must integrate seamlessly into existing workflows through vendor-neutral interoperability standards, automated DICOM processing, and intuitive reporting interfaces. Furthermore, models should demonstrate robust performance across diverse acquisition protocols, imaging devices, patient positioning, and common sources of image degradation such as motion artifacts or metallic implants, conditions that are often underrepresented in curated research datasets.
- **Regulatory approval and clinical validation**.
  Regulatory approval represents another critical step toward clinical implementation. None of the studies included in this review reported regulatory clearance, such as U.S. FDA authorization or European MDR certification. Under current regulatory frameworks, AI-based fracture detection systems are generally considered SaMD, requiring rigorous prospective clinical validation before approval (Zech et al., 2022; Elbahi et al., 2025). However, prospective validation was performed in only a very small proportion of the reviewed studies, highlighting a substantial gap between research prototypes and clinically deployable technologies.
- **Clinical governance and medico-legal responsibility**. The reviewed literature also provides limited guidance regarding clinical governance and legal accountability. None of the included studies explicitly addressed responsibility for diagnostic errors involving AI-assisted interpretations or described standardized human oversight procedures beyond indicating that AI should assist rather than replace clinicians. Safe implementation requires clearly defined governance structures specifying who reviews AI-generated findings, how disagreements between clinicians and algorithms are resolved, and how these decisions are documented. Such considerations become particularly important for uncommon but clinically significant injuries, including atypical femoral fractures and occult pelvic fractures, where missed diagnoses may have serious consequences.
- **User acceptance and reimbursement**. Long-term adoption of AI also depends on clinician acceptance and sustainable reimbursement models. None of the reviewed studies investigated reimbursement mechanisms for AI-assisted fracture interpretation or systematically evaluated user acceptance beyond reader-performance comparisons (Chen et al., 2021). Previous implementation studies have shown that clinicians are more likely to trust AI systems when predictions are accompanied by confidence estimates, interpretable explanations, and transparent descriptions of model limitations (Amann et al., 2020). In addition, successful implementation will require targeted training for radiologists and orthopedic surgeons, together with reimbursement frameworks that recognize the added clinical value of AI-assisted image interpretation.

Overall, the findings of this review suggest that the main obstacle preventing widespread clinical adoption of AI for atypical and complex fracture diagnosis is no longer limited to diagnostic performance alone. Rather, the literature reveals a striking lack of evidence regarding workflow integration, regulatory readiness, governance, reimbursement, and long-term implementation strategies. Since none of these dimensions were systematically evaluated across the included studies, future research should complement algorithm development with prospective implementation studies that assess the real-world feasibility, safety, and sustainability of AI-assisted fracture diagnosis.

### 4.4 Limitations of this review

This systematic review has several limitations. First, publication bias may have influenced the available evidence, as studies reporting positive AI performance are more likely to be published than those with negative or inconclusive findings. Second, only studies published in English were included. As a result, relevant studies published in other languages may have been missed. Third, the Google Scholar search was limited to the first 1843 records ranked by relevance. This approach was adopted because of the large number of retrieved records and the platform's ranking system. However, some eligible studies may not have been identified. Fourth, the included studies were highly heterogeneous. They differed in imaging modality, fracture type, AI architecture, clinical task, validation strategy, and performance metrics. Because of these differences, a quantitative meta-analysis was not appropriate. A structured narrative synthesis was therefore used instead. Finally, fractures were classified as “atypical” or “complex” using predefined operational criteria (Table 3). The classification was performed independently by two reviewers to improve consistency. However, some borderline cases may still have involved subjective judgment. These limitations should be considered when interpreting the findings of this review.

## 5 Conclusion

This review provides a comprehensive evaluation of the use of AI in the detection, classification, and segmentation of complex and atypical bone fractures using clinical imaging data. It focuses exclusively on studies employing AI techniques. To maintain clinical relevance, studies limited to simple fractures, synthetic datasets, or lacking verifiable outcomes were excluded. Findings indicate that hybrid architectures, combining convolutional networks with segmentation, detection, or classification sub-modules, can achieve strong performance for specific tasks, most consistently for pelvic multi-fragment fracture segmentation and vertebral fracture segmentation with classification, but their advantage over single-stage architectures is not uniform across anatomical regions or clinical tasks: for pelvic fracture detection and AO/OTA grading tasks, for example, hybrid pipelines achieved only moderate discrimination (AUC 0.74–0.89), while transformer-based and single-stage CNN approaches sometimes matched or exceeded hybrid performance for other fracture types (e.g., rib fracture detection with RetinaNet, sensitivity up to 97.1%). Preliminary evidence also suggests that multimodal fusion of imaging with electronic health record data can improve classification of atypical femoral fractures, increasing sensitivity from 79.6% to 90.3% and AUC from 0.966 to 0.987. However, this observation currently rests on a single research group's cohort and requires independent, multicenter replication before being generalized. These distinctions matter clinically: model selection should be guided by fracture type and task rather than by architectural category alone, and claims of AI readiness should be anchored to the specific anatomical and task context in which performance was demonstrated. Despite this potential, key limitations persist across the evidence base. The large majority of included studies (62.5%) relied on single- or bi-center, retrospectively validated data, with external validation reported in only 27.5% of studies and prospective validation in a single study (2.5%); standardized evaluation protocols were rarely applied, and MRI-based approaches remained almost entirely unexplored. These constraints, rather than architectural limitations alone, currently represent the principal barrier to broader generalizability and clinical integration. To move the field forward, future research must prioritize improving model interpretability, adopting unified evaluation standards, and validating AI systems across diverse imaging modalities and patient populations. A central challenge remains the development of models capable of handling highly complex and atypical fractures with the precision and nuance such cases demand. Equally essential is the availability of diverse, well annotated public datasets encompassing various fracture types, anatomical regions, and clinical contexts to ensure real-world applicability. Ultimately, AI should be viewed not as a replacement for radiologists, but as an intelligent assistive tool enhancing diagnostic accuracy, accelerating clinical decision making, and contributing to more personalized and effective patient care. Therefore, the current evidence supports further clinical investigation and prospective evaluation rather than indicating full clinical maturity or routine deployment of AI systems in fracture care.

## Glossary

2D/3D, Two-dimensional/Three-dimensional; AFF, Atypical Femoral Fracture; AI, Artificial Intelligence; AO/OTA, Arbeitsgemeinschaft für Osteosynthesefragen/Orthopedic Trauma Association; ASPP, Atrous Spatial Pyramid Pooling; AUC, Area Under the Curve; AUC-ROC, Area Under the Receiver Operating Characteristic Curve; CAM, Class Activation Map; CDDA, Cross-Dimensional Dual Attention; CNN, Convolutional Neural Network; COPD, Chron-ic Obstructive Pulmonary Disease; CXR, Chest X-ray; CT, Computed Tomography; DICOM, Digital Imaging and Communications in Medicine; DMF, Dual-Scale Multi-Feature Fusion; EBM, Evidence-Based Medicine; EHR, Electronic Health Record; FDD-Net, Fracture Detection and Delineation Network; FDA, Food and Drug Administration; FU-Net, Feature U-Net; GAC, Geodesic Active Contour; GCViT, Global Context Vision Transformer; GPU, Graphics Processing Unit; IoU, Intersection over Union; LMAF, Lateral Medial Anterior Fragment; MAPNet, Multi-scale Attention Pyramid Network; MDR, Medical Device Regulation; MeSH, Medical Subject Headings; MFFM, Multi-scale Feature Fusion Module; ML-KNN, Multi-Label k-Nearest Neighbors; MRI, Magnetic Resonance Imaging; MSDA-MLNet, Multi-Scale Dual-Attention Multi-Level Network; MULAN, Multi-task Learning for Analysis; mAP50, Mean Average Precision at IoU threshold 0.50; NASSS, Non-adoption, Abandonment, Scale-up, Spread, and Sustainability; NFF, Normal Femoral Fracture; nnU-Net, No-New-Net U-Net; PACS, Picture Archiving and Communication System; PICO, Population, Intervention, Comparison, Outcome; PRISMA, Preferred Reporting Items for Systematic Reviews and Meta-Analyses; QUADAS-2, Quality Assessment of Diagnostic Accuracy Studies 2; RE, Region Extraction (module); RIS, Radiology Information System; ROI, Region of Interest; SaMD, Software as a Medical Device; SCNN, Simple Convolutional Neural Network; SEG, Segmentation category; DET, Detection category; ENS, Ensemble category; HYB, Hybrid category; CLASSIC, Classical machine learning category; SFO, Simple Fracture Only; SSD, Single Shot MultiBox Detector; TRANS, Transformer-based category; UNETR, U-Net with Transformer Encoder; VCFs, Vertebral Compression Fractures; VFHA-CAM, Volume Feature Highlighting Attention Class Activation Map; ViT, Vision Transformer; YOLO, You Only Look Once; relevance-CAM, Relevance-weighted Class Activation Mapping.
