# Supplementary material for: Deep learning and hybrid architectures for atypical and complex bone fracture diagnosis: a systematic review of performance and clinical validity
Source: Front Artif Intell. 2026 Aug 10;9:1909177. doi: 10.3389/frai.2026.1909177 (PMC13500594; doi:10.3389/frai.2026.1909177)
Supplement: Supplementary file 1 [file Supplementary_File_1.pdf]

SUPPLEMENTARY MATERIAL

This supplementary file contains additional tables describing the search strategies, AI models, QUADAS-2 assessment criteria, clinical objectives, validation levels, and study design characteristics.

Supplementary Table S1. Search strategies used for the literature search

PubMed		Cochrane	
**#**	**Query**	**#**	**Query**
1	Fractures, Bone OR Femoral Fractures OR Osteoporotic Fractures OR Fractures, Comminuted OR Fracture Healing OR atypical fracture* OR complex fracture* OR nonunion fracture* OR occult fracture* OR bone stress injury OR insufficiency fracture* OR multifragmentary fracture* OR comminuted fracture* OR non-displaced fracture* OR fracture malunion* OR multiple fractures*	1	atypical fracture* OR complex fracture* OR comminuted fracture* OR fragility fracture* OR multiple fractures*
2	fracture detection OR image segmentation OR automated diagnosis OR deep learning OR CNN OR AI OR CT scan OR MRI imaging OR radiograph analysis OR diagnostic imaging	2	fracture detection* OR image segmentation* OR CNN OR deep learning* OR CT scan* OR MRI imaging*
3	#1 AND #2	3	advanced algorithm* OR machine learning* OR AI algorithm* OR automated method*
4	advanced algorithm* OR deep learning OR neural network* OR machine learning OR AI algorithm* OR CNN	4	traditional algorithm* OR classic method* OR conventional method*
5	#3 AND #4	5	Dice score* OR sensitivity OR specificity OR AUC-ROC
6	manual segmentation OR radiologist assessment OR traditional algorithm* OR semi-automated method*	6	#1 AND #2 AND #3 AND #5
7	Dice score OR sensitivity OR specificity OR processing time OR false negative rate OR AUC-ROC	7	#1 AND #2 AND #4 AND #5
8	#3 AND #6	8	#1 AND #2
9	#7 AND #8	9	Date filter: 2015–2026
10	English/French; 2015–2026	10	English/French

Google Scholar		IEEE Xplore	
**#**	**Query**	**#**	**Query**
1	bone fracture OR skeletal fracture OR hip fracture OR femoral fracture OR osteoporotic fracture OR fragility fracture OR atypical fracture*	1	atypical fracture* OR complex fracture* OR comminuted fracture* OR nonunion fracture* OR bone stress injury OR insufficiency fracture*
2	diagnostic imaging OR CT scan OR computed tomography OR MRI OR X-ray imaging OR image processing OR fracture detection	2	fracture detection OR image segmentation OR automated diagnosis OR CT scan OR MRI imaging OR radiograph analysis
3	artificial intelligence OR machine learning OR deep learning OR CNN OR computer vision	3	deep learning OR advanced AI models OR transformer-based models OR advanced segmentation
4	manual segmentation OR manual annotation OR radiologist assessment OR traditional algorithm*	4	traditional CNN models OR classical image analysis OR traditional algorithms
5	Dice score OR sensitivity OR specificity OR ROC curve OR AUC ROC	5	Dice score OR sensitivity OR specificity OR AUC-ROC
6	#1 AND #2 AND #3 AND #5	6	#1 AND #2 AND #3 AND #5
7	#1 AND #2 AND #4 AND #5	7	#1 AND #2 AND #4 AND #5
8	#6 OR #7	8	#1 AND #2
9	Filters: 2015–2026, English/French, patents excluded	9	Filters: 2015–2026, English/French
10	–	10	(#6 OR #7) AND filters

Supplementary Table S2. Summary of AI models for fracture detection, classification, and segmentation by type.

Tasks	Anatomy	AI model	Architecture category	Performance
Classification	Cranial	SkullNetV1 (CNN + ML-KNN)	HYB	Accuracy: 88%, F1: 93%, AUC: 0.89–0.98, Hamming Score: 92%, Hamming Loss: 0.04 [31]
Detection / Localization / Classification	Rib	RetinaNet+CNN	HYB	Sensitivity: 97.11% (right), 94.87% (left); F1: 0.872–0.971; AUC: 0.938–0.985; 12.6 s/case vs. 108 s for radiologists [28]
Detection & Segmentation	Rib	FracNet (3D U-Net)	SEG	Sensitivity: 92.9%, FP/scan: 5.27, Dice: 71.5%, IoU: 55.6% [57]
Segmentation	Rib	MAPNet	HYB	Acc: 88.06%, Sens: 89.26%, Spec: 87.58%, Dice: 85.41%, IoU: 80.37%; outperforms 3D U-Net [54]
Detection	Rib	CFSG U-Net	SEG	Sensitivity at FP rates 0.5–8/scan: 67.15–89.58%; Avg. FROC: 81.28% [59]
Segmentation & Classification	Vertebral	3D U-Net + freeze-and-grow	HYB	Dice: 0.984, Fracture Acc: 98.3%, Correct labeling: 100%, Low-dose Dice: 0.997 [53]
Segmentation	Vertebral	FU-Net	HYB	Dice: 96.4% / 92.8% (non-fractured/fractured); 95% (CSI 2016/xVertSeg.v1) [9]
Detection / Quantification	Vertebral	Anchor-free CNN	HYB	Dice: 0.99, Precision: 0.99, Recall: 0.99, AUC: 0.96; 2 s/CT [30]
Segmentation	Complex	DeepLabV3 + MobileNetV3	SEG	Dice: 84.92%, IoU: 93.16% (Val) / 96.46% (Train), AUC: 0.99, Inference: 0.005 s [55]
Detection	Humerus	YOLOv4	DET	mAP: 63.5% (YOLOv3: 48.4%) [69]
Classification	Ankle	ResNet	SCNN	AUC: 0.90; Type A: 0.81, B: 0.93, C: 0.86; comparable to experts ($\kappa = 0.55$) [58]
Classification AFF vs. NFF	Femur	ResNet50 + Dense Network	HYB	Sensitivity: 79.6%–90.3%, AUC: 0.966–0.987 [60]
Classification AFF vs. NFF	Femur	ResNet50	SCNN	AUC: 0.89–0.94, Sensitivity: 89.0%–94.0% [12]
Classification	Femoral	DenseNet-172	SCNN	AUC: 99.4% (radiologists: 96.9%), external AUC: 98.0% [56]
Classification Pelvic	Pelvis	Tri-planar ResNeXt50	ENS	Acc: 55.8–85.0%, Sens: 37.6–75.7%, Spec: 71.7–96.5%, F1: 0.43–0.77 [29]
Detection	Pelvis	Multi-orientated YOLOv3	ENS	AUC: 0.824, Precision: 90.7%, Recall: 80.5% [36]
Prediction	Pelvis	Boring-survey + 3D-CNN	HYB	Acc: 79.7%,Recall: 80.6%, Precision: 60.0% [37]
Segmentation + Classification	Pelvic	FDD-Net + DMF	HYB	Dice: 0.935–0.955, Jaccard: 0.891–0.918, Sens: 0.912–0.929, HD95: 0.770–0.937 mm [16]
Semantic Segmentation	Tibial	DeepLab v3+ (ResNet-50)	SEG	Global Acc: 98.7%, Weighted IoU: 0.9841, Boundary F1: 0.8921 [72]
Detection	Facial	VFHA-CAM	DET	Sens: 87%, Spec: 85%, Analysis time: 76s (15× faster than standard CAM) [35]
Detection	Nasal	YOLOX + GhostNetv2	HYB	Acc: 97.44%, Sens: 95%, Spec: 95.24% [52]
Classification	Cervical	ResNet50V2 / VGG16	SCNN	Acc: 92%, F1-score: [0.88–0.93], Recall (at Precision 0.8)[38]
Multi-label Cls. + Detection	Cervical spine	GCViT + YOLOv8	HYB	Macro F1: 0.96, EMR: 0.95 — mAP50: 0.96, mAP50-95: 0.747 [70]
Segmentation	Fractured radius	3D-GAC	CLASSIC	Dice: 99.2%, Failure rate: 8%, Contour error $<$1.3%,Fracture failure rate: 8.3% (5/60) [73]
Multi-class Cls. + instance segmentation	Distal radius (AO/OTA)	Mask R-CNN (ResNet-50)	HYB	Acc: 61–62%, AUC: 0.56–0.93; (2R3A: 0.82–0.84, 2R3B: 0.56–0.63, 2R3C: 0.75) [65]
Binary Classification	Hairline	iA-HLD (AlexNet)	SCNN	Acc: 97.6%, Precision/Recall/F1: 98% [10]
Detection	Rib / Clavicle	CXR-FxNet	DET	AUC: 91.2%, Acc: 83.3%, Sens: 87.5%, , Sens: 87.5%, Spec: 79.1% [67]
Classification	VCFs	MULAN	HYB	Sensitivity: 0.770, Specificity: 0.981; image-level labels only [61]
Det. + Class. + Seg.	Monteggia	U-Net++ + ResNet50 + EfficientNet-B1	HYB	Dice: 0.878–0.912, IoU: 0.794–0.852; Sens: 92.3–100%, Spec: 96.3–96.9% (dislocation) ; AUC: 0.923, Acc: 85.7 % (fracture ulnaire)[76]
detection+classificatio	Pelvic (various)	Faster-RCNN + Bayesian	HYB	AUC: 0.74–0.89; interpretable; comparable to radiologists [77]
Hybrid (Segmentation + Classification)	Thoracolumbar CT	3D V-Net + ResNet18	HYB	Seg. DSC: 0.90 — Cls. AUC: 0.87–0.96, Acc: 93% [74]
Class. + Seg.	Vertebral fractures	nnU-Net + ResNet-18	HYB	Sens: 87.9%, Spec: 94.9%, Acc: 93.0% [78]
Classification	Pathologic femur	ViT-B/16 + DINO	HYB	Acc: 0.848–0.880; AUROC: 0.928–0.947; outperforms surgeons [64]
Detection + Classification	Fresh VCFs	EfficientNet, MobileNet, MnasNet	SCNN	AUC: 0.815–0.824 (Control); Acc: 78.1–80.7% (Control); outperforms surgeons [62]
Detection + Classification	LMAF vs. SFO	YOLOv12, Faster R-CNN, SSD, RetinaNet	DET	YOLOv12 mAP50: 92.1%, AUC: 0.983; others mAP50: 63–67% [68]
Detection & CWIS Classification	Ribs	3× nnDetection + 1× nnU-Net	HYB	Detection Sens: 80–84%, Prec: 85–87%; Rib Numbering Acc: 94–95% [63]
Multi-fragment Segmentation (2D/3D)	Pelvis	U-Net (ResNet50/VGG19), LinkNet, UNETR	SEG	2D Dice: 0.991, IoU: 0.982; 3D Dice: 0.844–0.911, Spec: 0.993, Sens: 0.730[75]
Opportunistic Detection & Classification	Vertebrae	IB Lab FLAMINGO (Sequential: Identification + CNN Ensemble)	HYB	Acc: 0.93, Sens: 0.86, Spec: 0.99[82]
Detection & Classification	Tibial plateau	GoogleNet, ResNet	SCNN	Detection - Sens: 92.7%, Acc: 70.4%, F1: 0.76; Classification - Acc: 34.6%, Sens: 32.1% [71]
Classification	Cervicothoracic spine (C7–T1)	MSDA-MLNet (MFFM + CDDA)	SCNN	Acc: 91.82%, F1: 91.24%, +9.78% accuracy improvement, 37.05% reduced training time [66]

Supplementary Table S3. Extended AI-adapted QUADAS-2 tool: operational criteria for risk of bias and applicability assessment (0–3 scale)

**Domain**	**0 – Low risk**	**1 – Moderate risk**	**2 – High risk**	**3 – Unclear/unreported**
Patient Selection	Prospective or well-justified multicenter recruitment; clear, transparent inclusion/exclusion criteria; no post-hoc exclusion	Retrospective, keyword-based or pre-existing/public cohort, but multicenter or transparent inclusion criteria	Single-center retrospective cohort with unclear recruitment strategy (consecutive/random/opportunistic not specified) or post-hoc anatomical exclusion	Recruitment strategy and inclusion/exclusion criteria not reported
Index Test (AI model)	Independent train/validation/test split (patient-level); predefined hyperparameters; external validation reported; fully automated pipeline	Small dataset without cross-validation, or human-in-the-loop refinement, but methodology described and thresholds objectively set (e.g., Youden index)	Data-splitting/validation strategy poorly described; risk of data leakage; arbitrary post-processing tuned on the same dataset; no external validation	Validation strategy not described (e.g., only "train/test split" mentioned, no validation set)
Reference Standard	Multiple experienced annotators with blinded consensus review; standardized criteria (e.g., Genant)	Single annotation platform or human-in-the-loop workflow with possible confirmation bias; reused public annotations without independent re-verification	Annotations by a single radiologist without independent double reading or consensus adjudication	Annotation process/reference standard not described
Flow and Timing	Identical protocol for all patients; natural prevalence preserved; index test and reference standard applied to the same examinations	Fracture-positive-only inclusion or separate datasets per sub-task, without evidence of prevalence manipulation	Artificial class rebalancing (e.g., forced positive/negative ratio) or heavy synthetic augmentation altering natural case distribution	Provenance/allocation of added or excluded cases between train and test not reported
Applicability – Patient	Study population directly matches the intended real-world target population, representative demographics	Population narrower than target use case (e.g., restricted protocol, single anatomical subtype) but still broadly relevant	Population drawn from an atypical clinical subgroup (e.g., disease-specific cohort) not generalizable to the general screening population	Population characteristics not reported in sufficient detail
Applicability – Index Test	Operates on standard DICOM images; fully automated; compatible with routine PACS workflow; interpretable output	Requires non-standard preprocessing or partial manual steps	Substantial technical constraints reported (e.g., low-end hardware trade-offs, coarse/non-clinical output)	Workflow compatibility not discussed
Applicability – Reference Standard	Reference standard matches exactly the diagnostic target needed for clinical/surgical decision-making	Reference standard partially aligned with clinical need, minor scope mismatch	Reference standard reflects a narrower or different construct than the clinical target	Insufficient information to assess alignment with clinical practice

This is a provisional file, not the final typeset article

Supplementary Table S4. Classification of AI models for fracture imaging according to their primary clinical objective

Clinical objective	Representative architectures (Ref.)	Main strengths / Clinical output	Main limitations
**Classification** (15 studies)	ML-KNN [31]; MSDA-MLNet [66]; GoogleNet/ResNet [71]; EfficientNet/MobileNet/MnasNet [62]; ViT-B/16+DINO [64]; MULAN [61]; RetinaNet [28]; modified ResNet [58]; ResNet50+DenseNet [60]; ResNet50 [12]; DenseNet-172 [56]; ResNeXt50 [29]; YOLOX+GhostNetv2 [52]; ResNet50V2/VGG16 [38]; iA-HLD/AlexNet [10]	Automated fracture classification and severity grading (AO/OTA, Schatzker) [29, 58]; diagnostic performance comparable or superior to some clinicians [56, 62]; multimodal integration of imaging and clinical data supporting differential diagnosis [60]; robustness to class imbalance using image-level labels [61]; improved performance of junior and senior clinicians [52]; anatomy-independent fracture recognition [10].	High inter-observer variability in reference annotations, particularly for complex classifications [71]; manual ROI selection and absence of external validation in several studies [62]; single-center or single-radiologist datasets [61]; inability to evaluate associated soft-tissue injuries [58]; performance degradation on external cohorts [56, 64]; limited sample size and restricted generalizability [52, 10].
**Detection** (8 studies)	YOLOv12 [68]; CFSG U-Net [59]; anchor-free 2D keypoint detector [30]; YOLOv4 [69]; multi-oriented YOLOv3 [36]; CXR-FxNet [67]; BSFD+3D-CNN [37]; 3D RetinaNet+VFHA-CAM [35]	Automated spatial localization using bounding boxes, keypoints or activation maps [35, 68]; improved diagnostic performance compared with radiologists in specific tasks [68]; interpretable localization with robustness to severe scoliosis [30]; simultaneous detection of rib and clavicle fractures on standard radiographs [67]; 3D fracture localization without manual voxel-level annotation and faster processing than conventional CAM approaches [35].	Dependence on manually annotated bounding boxes or keypoints [68]; increased false-positive rates and absence of external validation [59]; coarse localization without fragment characterization [69]; reduced sensitivity for specific fracture patterns such as compression fractures [36]; limited expert annotations and reduced external performance [67]; small monocentric cohorts [37].
**Segmentation** (7 studies)	3D U-Net/MAPNet/FU-Net/DeepLabV3+/3D-GAC/UNETR comparison [75]; FracNet [57]; MAP-Net [54]; FU-Net level-set [9]; DeepLabV3+MobileNetV3 [55]; DeepLabV3+ResNet50 [72]; 3D-GAC [73]	Precise voxel-level fracture delineation enabling automated volume measurement [57]; preservation of anatomical structures with efficient projection-based processing [54]; quantitative assessment of fracture fragments; management of complex fragmentation with up to 23 fragments and reduced analysis time compared with experts [72]; robustness to cortical disruption and casts validated by expert readers [73].	Large manual annotation requirements and risk of overfitting [75]; reduced overlap for thin and elongated fracture patterns [57, 54]; sensitivity to optimization parameters in level-set approaches [9]; absence of external validation and possible bone overestimation [72]; reported failure rate for specific fracture configurations [73].
**Hybrid**			

Continued on next page

**Clinical objective**	**Representative architectures (Ref.)**	**Main strengths / Clinical output**	**Main limitations**
(10 studies; composite output)	3D V-Net+ResNet18 [74]; FLAMINGO [82]; 3D U-Net+freeze-and-grow [53]; Faster R-CNN+Bayesian model [77]; Pelvic FDD-Net+DMF [16]; GCViT+YOLOv8 [70]; Mask R-CNN [65]; U-Net+++ResNet50+EfficientNet-B1 [76]; nnU-Net+ResNet18 [78]; multi-stage nnDetection+nnU-Net [63]	Integrated clinical pipelines combining detection, classification, segmentation and anatomical localization [65, 63]; superior performance compared with experienced clinicians with multicenter and prospective validation [74]; Bayesian correction module improving recovery of false negatives [77]; multicenter external validation and open-source implementation [65]; PACS integration and longitudinal reporting capability [78]; automated fracture detection, classification and rib numbering with external validation [63].	Limited training cohorts despite complex pipelines [74, 78]; single-center validation in some studies [82]; absence of independent external testing in some approaches [77]; potential propagation of errors between sequential modules [16]; limited direct comparison with clinicians [70, 76]; severe class imbalance and long inference time for complex 3D pipelines (20–90 min) [63].

This is a provisional file, not the final typeset article

Supplementary Table S5. Clinical validation level assessment of the included AI studies.

Task	Modality	Fracture type	Validation level	Readiness tier
Multi-label classification	CT	Linear, depressed, linear non-depressed, facial, and comminuted	Internal validation, bi-center dataset	Tier 1 [31]
Multi-task (Detection and Classification)	CT	Rib (fresh, healing, and old fractures, including subtle presentations)	Multicenter, internal and external	Tier 3 [28]
Detection + Segmentation	CT	Rib (traumatic)	Internal, single-center, held-out test set	Tier 1 [57]
Segmentation	CT	Rib	Internal, expert-annotated multicenter dataset	Tier 1 [54]
Detection and segmentation	CT	Rib	Internal only, no external validation	Tier 1 [59]
Segmentation + classification	CT	Thoracolumbar spine	Internal validation with generalizability testing	Tier 2 [53]
Segmentation	CT	Osteoporotic vertebral	Internal validation only	Tier 1 [9]
Detection / quantification	CT	Vertebral	External validation	Tier 3 [30]
Segmentation	X-ray	Appendicular and axial skeleton / mixed	Internal benchmark	Tier 1 [55]
Detection	X-ray	Humeral	Internal validation (MURA split)	Tier 1 [69]
Hierarchical classification	X-ray	Ankle	Internal, single-center, reader comparison	Tier 2 [58]
Multimodal binary classification	X-ray + clinical data	AFF vs. NFF	Internal validation, multicenter (72 hospitals)	Tier 1 [60]
Binary image classification	Plain X-ray	AFF vs. NFF	Internal validation (5-fold cross-validation)	Tier 1 [12]
Binary image classification	X-ray	Proximal femoral / hip fracture	External validation (international, multicenter)	Tier 3 [56]
Multi-class image classification	CT	Pelvic	Internal validation (5-fold CV, multi-center data)	Tier 2 [29]
Detection	3D CT	Pelvis	Internal validation (6-fold CV, separate control cohort), monocenter	Tier 1 [36]
Multimodal classification + 3D prediction	PXR + CT	Pelvic ring, hip, acetabular, and FFP	Internal validation, patient-level isolation	Tier 1 [37]
Segmentation	CT	Complex pelvic ring disruptions	External validation, multicenter independent benchmarking	Tier 3 [16]
Multi-class 2D/3D semantic segmentation	CT	Comminuted tibia and fibula	Internal validation + Reader comparison	Tier 2[72]
Detection and segmentation	3D-CT	Complex facial bone fractures	Internal validation + Clinical software integration	Tier 2 [35]
Detection + binary classification	CT	Nasal	Internal validation + + Reader comparison	Tier 2 [52]
Detection and classification	X-ray	Cervical	Internal validation only	Tier 1 [38]
Multi-label Cls. + Detection	CT	Cervical spine	Multi-institutional training set, no independent external test set	Tier 1 [70]
3D segmentation + active contours	HR-pQCT	Distal radius	Internal validation supplemented by a 3-reader expert comparison benchmark	Tier 2 [73]

Continued on next page

**Task**	**Modality**	**Fracture type**	**Validation level**	**Readiness tier**
Multi-class Cls. + instance segmentation	X-ray	Distal radius	External geographical validation (intercontinental)	Tier 3 [65]
Binary classification	X-ray	Hairline / stress fractures	Internal validation with test-time augmentation	Tier 1 [10]
Detection and localization	Chest X-ray	Rib and clavicle fractures	External geographical validation	Tier 3 [67]
WS multi-label Cls. + Loc.	CT	Vertebral (VCFs)	Internal, independent test set (single hospital)	Tier 1 [61]
Segmentation + quantification + classification	X-ray	Pediatric Monteggia	Internal, independent test set	Tier 1 [76]
Detection	CT	Pelvic ring disruptions (Tile AO/OTA)	Multicenter, 5-fold cross-validation (no independent external test set)	Tier 2 [77]
Segmentation and binary classification	CT	Thoracolumbar vertebral	External, geographic and temporal, prospective validation	Tier 3 [74]
Segmentation and binary classification	CT	Lumbar/Thoracolumbar spine	Internal validation, independent retrospective test set, reader comparison	Tier 2 [78]
Classification	X-ray	Pathologic proximal femur	Multicenter, internal expert benchmark, external validation	Tier 3 [64]
Classification	X-ray	Fresh vertebral (VCFs)	Internal validation, cross-validation, reader comparison	Tier 2 [62]
Detection + classification	CT	Ankle / lateral malleolus	External, multi-institutional validation	Tier 3 [68]
Detection + CWIS classification	CT	Rib	Multicenter, external validation	Tier 3 [63]
Semantic segmentation	CT	Pelvic (multi-fragment)	Internal, 5-fold CV, patient-level split, no external validation	Tier 1 [75]
Detection + classification	CT	Fragility vertebral	Independent external validation	Tier 3 [82]
Detection / classification	X-ray	Tibial plateau	Multicenter Internal Validation	Tier 2 [71]
Classification	CT	Cervicothoracic spine	Internal validation only	Tier 1 [66]

Supplementary Table S6. Study design and validation characteristics of included AI-based fracture detection studies.

Reference	Design	Multi-center	Internal validation	External validation	Independent test set	Clinician comparison
Emon et al. (2022) [31]	Retro	Yes	Yes	No	No	No
Zhou et al. (2022) [28]	Retro	Yes	Yes	Yes	Yes	Yes
Jin et al. (2020) [57]	Retro	No	Yes	No	Yes	Yes
Gao et al. (2022) [54]	Retro	Yes	Yes	No	Yes	No
Gao et al. (2022) [59]	Retro	No	Yes	No	Yes	No
Nadeem et al. (2024) [53]	Retro	Yes	Yes	No	Yes	Yes
Rehman et al. (2019) [9]	Retro	Yes*	Yes	No	Yes	Yes
Zakharov et al. (2022) [30]	Retro	Yes	Yes	Yes	Yes	Yes
Turk et al. (2024) [55]	Retro	Yes	Yes	No	Yes	No
Verma et al. (2024) [69]	Retro	No	Yes	No	Yes	No
Olczak et al. (2020) [58]	Retro	No	Yes	No	Yes	Yes
Schilcher et al. (2024) [60]	Retro	Yes	Yes	No	Yes	No
Zdolsek et al. (2021) [12]	Retro	No	Yes	No	No	No
Oakden et al. (2022) [56]	Retro	Yes	Yes	Yes	Yes	Yes
Dreizin et al. (2021) [29]	Retro	Yes	Yes	No	Yes	Yes
Ukai et al. (2021) [36]	Retro	No	Yes	No	Yes	No
Rahman et al. (2024) [37]	Retro	No	Yes	No	Yes	No
Zeng et al. (2024) [16]	Retro	Yes	Yes	Yes	Yes	No
Kim et al. (2023) [72]	Retro	No	Yes	No	Yes	Yes
Moon et al. (2024) [35]	Retro	No	Yes	No	Yes	Yes
Wang et al. (2024) [52]	Retro	No	Yes	No	Yes	Yes
Meadi et al. (2024) [38]	Retro	NR	Yes	No	Yes	No
Behbahani Nejad et al. (2023) [70]	Retro	Yes	Yes	No	Yes	No
Ohs et al. (2021) [73]	Retro	No	Yes	No	Yes	Yes
Nijhuis et al. (2025) [65]	Retro	Yes	Yes	Yes	Yes	Yes
Jain et al. (2024) [10]	Retro	Yes†	Yes	No	Yes	No
Cheng et al. (2024) [67]	Retro	Yes	Yes	Yes	Yes	Yes
Choi et al. (2023) [61]	Retro	No	Yes	No	Yes	No
Chakladar et al. (2026) [76]	Retro	No	Yes	No	Yes	No
Chen et al. (2025) [77]	Retro	Yes	Yes	No	No	Yes
Chen et al. (2025) [74]	Mixed	Yes	Yes	Yes	Yes	Yes
Glessgen et al. (2025) [78]	Retro	No	Yes	No	Yes	Yes
Kim et al. (2021) [64]	Retro	Yes	Yes	Yes	Yes	Yes
Li et al. (2025) [62]	Retro	Yes	Yes	No	Yes	Yes
Liu et al. (2025) [68]	Retro	Yes	Yes	Yes	Yes	Yes
Marting et al. (2025) [63]	Retro	Yes	Yes	Yes	Yes	Yes
Nesheli et al. (2026) [75]	Retro	Yes	Yes	No	Yes	No
Spångeus et al. (2025) [82]	Retro	No	Locked software	Yes	Yes	Yes
van der Gaast et al. (2025) [71]	Retro	Yes	Yes	No	Partial	No
Yan et al. (2025) [66]	Retro	No	Yes	No	Partial	No

* Multi-center status reported with limited information. † Multi-center status inferred from study description. NR: not reported.

REFERENCES

[1]T. Meena and S. Roy, ”Bone fracture detection using deep supervised learning from radiological images: A paradigm shift,” *Diagnostics (Basel)*, vol. 12, no. 10, p. 2420, Oct. 2022, doi: 10.3390/diagnostics12102420.

[2]S. Wang, G. Cao, Y. Wang, S. Liao, Q. Wang, J. Shi, C. Li, and D. Shen, ”Review and prospect: artificial intelligence in advanced medical imaging,” *Front. Radiol.*, vol. 1, p. 781868, 2021, doi: 10.3389/fradi.2021.781868.

[3]E. J. Topol, ”High-performance medicine: the convergence of human and artificial intelligence,” *Nat. Med.*, vol. 25, no. 1, pp. 44–56, 2019, doi: 10.1038/s41591-018-0300-7.

[4]A. Hosny, C. Parmar, J. Quackenbush, L. H. Schwartz, and H. J. W. L. Aerts, ”Artificial intelligence in radiology,” *Nat. Rev. Cancer*, vol. 18, no. 8, pp. 500–510, 2018, doi: 10.1038/s41568-018-0016-5.

[5]G. Litjens, T. Kooi, B. E. Bejnordi, A. A. A. Setio, F. Ciompi, M. Ghafoorian, J. A. W. M. van der Laak, B. van Ginneken, and C. I. Sánchez, ”A survey on deep learning in medical image analysis,” *Med. Image Anal.*, vol. 42, pp. 60–88, 2017, doi: 10.1016/j.media.2017.07.005.

[6]Z. Su, A. Adam, M. F. Nasrudin, M. Ayob, and G. Punganan, ”Skeletal fracture detection with deep learning: A comprehensive review,” *Diagnostics*, vol. 13, no. 20, p. 3245, 2023.

[7]P. H. S. Kalmet, S. Sanduleanu, S. Primakov, G. Wu, A. Jochems, T. Refaee, A. Ibrahim, L. v. Hulst, P. Lambin, and M. Poeze, ”Deep learning in fracture detection: a narrative review,” *Acta Orthop.*, vol. 91, no. 2, pp. 215–220, 2020, doi: 10.1080/17453674.2019.1711323.

[8]M. Kutbi, ”Artificial intelligence-based applications for bone fracture detection using medical images: a systematic review,” *Diagnostics*, vol. 14, no. 17, p. 1879, 2024, doi: 10.3390/diagnostics14171879.

[9]F. Rehman, S. I. A. Shah, M. N. Riaz, S. O. Gilani, and F. R., ”A Region-Based Deep Level Set Formulation for Vertebral Bone Segmentation of Osteoporotic Fractures,” J. Digit. Imaging, vol. 33, no. 1, pp. 191–203, 2020, doi: 10.1007/s10278-019-00216-0.

[10]B. Jain, D. Malik, G. Jagota, and I. Chandra, ”iA-HLD: An improved AlexNet for hairline fracture detection in orthopedic images,” *Neural Comput. Appl.*, vol. 36, pp. 23031–23047, 2024, doi: 10.1007/s00521-024-10348-2.

[11]L. M. Fayad, I. R. Kamel, S. Kawamoto, D. A. Bluemke, F. J. Frassica, and E. K. Fishman, ”Distinguishing stress fractures from pathologic fractures: A multimodality approach,” *Skeletal Radiol.*, vol. 34, no. 5, pp. 245–259, 2005, doi: 10.1007/s00256-005-0918-1.

[12]G. Zdolsek, Y. Chen, H.-P. Bögl, C. Wang, M. Woisetschläger, and J. Schilcher, ”Deep neural networks with promising diagnostic accuracy for the classification of atypical femoral fractures,” *Acta Orthop.*, vol. 92, no. 4, pp. 394–400, 2021, doi: 10.1080/17453674.2021.1891512.

[13]Y. Oh, K. Yamamoto, T. Yoshii, M. Kitagawa, and A. Okawa, ”Current concept of stress fractures with an additional category of atypical fractures: a perspective review with representative images,” *Ther. Adv. Endocrinol. Metab.*, vol. 12, p. 20420188211049619, 2021, doi: 10.1177/20420188211049619.

[14]A. A. Khan, R. H. J. A. Slart, D. S. Ali, O. Bock, J. J. Carey, P. Camacho, K. Engelke, P. A. Erba, N. C. Harvey, W. F. Lems, et al., ”Osteoporotic fractures: diagnosis, evaluation, and significance from the International Working Group on DXA best practices,” in *Mayo Clin. Proc.*, 2024, pp. 1127–1141, doi: 10.1016/j.mayocp.2024.01.011.

[15]M. H. Scheinfeld, A. A. Dym, M. Spektor, L. L. Avery, R. J. Dym, and D. F. Amanatullah, ”Acetabular fractures: What radiologists should know and how 3D CT can aid classification,” *Radiographics*, vol. 35, no. 2, pp. 555–577, 2015, doi: 10.1148/rg.352140098.

[16]B. Zeng, H. Wang, L. Joskowicz, and X. Chen, ”Fragment distance-guided dual-stream learning for automatic pelvic fracture segmentation,” *Comput. Med. Imaging Graph.*, vol. 116, p. 102412, Sep. 2024, doi: 10.1016/j.compmedimag.2024.102412.

[17]Y. Liu, S. Yibulayimu, G. Zhu, C. Shi, C. Liang, C. Zhao, X. Wu, Y. Sang, and Y. Wang, ”Automatic pelvic fracture segmentation: a deep learning approach and benchmark dataset,” *Front. Med.*, vol. 12, p. 1511487, 2025, doi: 10.3389/fmed.2025.1511487.

[18]Y. Zhou, X. Zou, and Y. Wang, ”Towards cross-scale attention and surface supervision for fractured bone segmentation in CT,” in *2024 46th Annu. Int. Conf. IEEE Eng. Med. Biol. Soc. (EMBC)*, 2024, pp. 1–5, doi: 10.1109/EMBC53108.2024.10781758.

[19]L. Chen, S. Li, Q. Bai, J. Yang, S. Jiang, and Y. Miao, ”Review of image classification algorithms based on convolutional neural networks,” *Remote Sens.*, vol. 13, no. 22, p. 4712, 2021, doi: 10.3390/rs13224712.

[20]Y. Yu, C. Wang, Q. Fu, R. Kou, F. Huang, B. Yang, T. Yang, and M. Gao, ”Techniques and challenges of image segmentation: A review,” *Electronics*, vol. 12, no. 5, p. 1199, 2023, doi: 10.3390/electronics12051199.
[21]G. Wood, K. M. Knapp, B. Rock, C. Cousens, C. Roobottom, and M. R. Wilson, ”Visual expertise in detecting and diagnosing skeletal fractures,” *Skelet. Radiol.*, vol. 42, pp. 165–172, 2013, doi: 10.1007/s00256-012-1503-5.
[22]J. M. Lee, J. Y. Park, Y. J. Kim, and K. G. Kim, ”Deep-learning-based pelvic automatic segmentation in pelvic fractures,” *Sci. Rep.*, vol. 14, no. 1, p. 12258, 2024, doi: 10.1038/s41598-024-63093-w.
[23]T. Aldhyani, Z. A. Ahmed, B. M. Alsharbi, S. Ahmad, M. H. Al-Adhaileh, A. H. Kamal, M. Almaiah, and J. Nazeer, ”Diagnosis and detection of bone fracture in radiographic images using deep learning approaches,” *Front. Med.*, vol. 11, p. 1506686, 2025, doi: 10.3389/fmed.2024.1506686.
[24]I. Abedeen, M. A. Rahman, F. Z. Prottyasha, T. Ahmed, T. M. Chowdhury, and S. Shatabda, ”Fracatlas: A dataset for fracture classification, localization and segmentation of musculoskeletal radiographs,” *Sci. Data*, vol. 10, no. 1, p. 521, 2023, doi: 10.1038/s41597-023-02432-4.
[25]X. Liu, H. Wang, T. Zhang, Y. Wan, and C. Zhang, ”Comparison between computed tomography and magnetic resonance imaging in clinical diagnosis and treatment of tibial platform fractures,” *World J. Clin. Cases*, vol. 8, no. 18, p. 4067, 2020, doi: 10.12998/wjcc.v8.i18.4067.
[26]X. Zhang, Y. Wang, C.-T. Cheng, L. Lu, A. P. Harrison, J. Xiao, C.-H. Liao, and S. Miao, ”A new window loss function for bone fracture detection and localization in X-ray images with point-based annotation,” arXiv:2012.04066, 2020, doi: 10.48550/arXiv.2012.04066.
[27]M. Jarraya, D. Hayashi, F. W. Roemer, M. D. Crema, L. Diaz, J. Conlin, M. D. Marra, N. Jomaah, and A. Guermazi, ”Radiographically occult and subtle fractures: a pictorial review,” *Radiol. Res. Pract.*, vol. 2013, p. 370169, 2013, doi: 10.1155/2013/370169.
[28]Q.-Q. Zhou, Z.-C. Hu, W. Tang, B. Zhang, L. Lu, and H. Zhang, ”Precise anatomical localization and classification of rib fractures on CT using a convolutional neural network,” *Clin. Imaging*, vol. 81, pp. 24–32, 2022, doi: 10.1016/j.clinimag.2021.09.016.
[29]D. Dreizin, F. Goldmann, C. LeBedis, A. Boscak, M. Dattwyler, U. Bodanapally, G. Li, S. Anderson, A. Maier, and M. Unberath, ”An automated deep learning method for Tile AO/OTA pelvic fracture severity grading from trauma whole-body CT,” *J. Digit. Imaging*, vol. 34, pp. 53–65, 2021, doi: 10.1007/s10278-020-00399-x.
[30]A. Zakharov, M. Pisov, A. Bukharaev, A. Petraikin, S. Morozov, V. Gombolevskiy, and M. Belyaev, ”Interpretable vertebral fracture quantification via anchor-free landmarks localization,” *Med. Image Anal.*, vol. 83, p. 102646, 2023, doi: 10.1016/j.media.2022.102646.
[31]M. M. Emon, T. R. Ornob, and M. Rahman, ”Classifications of skull fractures using CT scan images via CNN with lazy learning approach,” *J. Comput. Sci.*, vol. 18, no. 3, pp. 116–129, 2022, doi: 10.3844/jcssp.2022.116.129.
[32]T. D. Pham, S. B. Holmes, and P. Coulthard, ”A review on artificial intelligence for the diagnosis of fractures in facial trauma imaging,” *Front. Artif. Intell.*, vol. 6, p. 1278529, 2024, doi: 10.3389/frai.2023.1278529.
[33]J. Jung, J. Dai, B. Liu, and Q. Wu, ”Artificial intelligence in fracture detection with different image modalities and data types: A systematic review and meta-analysis,” *PLOS Digital Health*, vol. 3, no. 1, pp. e0000438, 2024.
[34]M. C. L. van den Broek, J. H. Buijs, L. F. M. Schmitz, and M. M. E. Wijffels, ”Diagnostic performance of artificial intelligence in rib fracture detection: Systematic review and meta-analysis,” *Surgeries*, vol. 5, no. 1, pp. 24–36, 2024.
[35]G. Moon, D. Lee, W. J. Kim, Y. Kim, K. Y. Sung, and H.-S. Choi, ”Very fast, high-resolution aggregation 3D detection CAM to quickly and accurately find facial fracture areas,” *Comput. Methods Programs Biomed.*, vol. 256, p. 108379, 2024, doi: 10.1016/j.cmpb.2024.108379.
[36]K. Ukai, R. Rahman, N. Yagi, K. Hayashi, A. Maruo, H. Muratsu, and S. Kobashi, ”Detecting pelvic fracture on 3D-CT using deep convolutional neural networks with multi-orientated slab images,” *Sci. Rep.*, vol. 11, p. 11716, 2021, doi: 10.1038/s41598-021-91246-z.
[37]M. R. Rahman, ”Advancing deep-learning-based fracture diagnosis in pelvic imaging with multimodal images,” Ph.D. dissertation, Graduate School of Engineering, Univ. of Hyogo, 2024.
[38]M. N. Meadi, A. E. M. Zerari, and H. Benbrahim, ”Cervical spine fracture detection using deep learning algorithm,” in *2024 8th Int. Conf. Image Signal Process. Appl. (ISPA)*, Biskra, Algeria, Apr. 21–22, 2024, pp. 1–8, doi: 10.1109/ISPA59904.2024.10536832.

[39]A. Paez, ”Gray literature: An important resource in systematic reviews,” *J. Evid. Based Med.*, vol. 10, no. 3, pp. 233–240, 2017, doi: 10.1111/jebm.12266.
[40]M. E. Falagas, E. I. Pitsouni, G. A. Malietzis, and G. Pappas, ”Comparison of PubMed, Scopus, web of science, and Google scholar: strengths and weaknesses,” *FASEB J.*, vol. 22, no. 2, pp. 338–342, 2008, doi: 10.1096/fj.07-9492LSF.
[41]A. Guni, V. Sounderajah, P. Whiting, P. Bossuyt, A. Darzi, H. Ashrafian, et al., ”Revised tool for the quality assessment of diagnostic accuracy studies using AI (QUADAS-AI): protocol for a qualitative study,” *JMIR Res. Protoc.*, vol. 13, no. 1, p. e58202, 2024, doi: 10.2196/58202.
[42]Veritas Health Innovation, ”Covidence Systematic Review Software,” Melbourne, Australia: Veritas Health Innovation, 2014. [Online]. Available: `https://www.covidence.org`
[43]D. R. Sarvamangala and R. V. Kulkarni, ”Convolutional neural networks in medical image understanding: A survey,” *Evolutionary Intelligence*, vol. 15, no. 1, pp. 1–22, Springer, 2022.
[44]M. Baumgartner, P. F. Jäger, F. Isensee, and K. H. Maier-Hein, ”nnDetection: A self-configuring method for medical object detection,” in *Medical Image Computing and Computer-Assisted Intervention (MICCAI 2021)*, Lecture Notes in Computer Science, vol. 12905, pp. 530–539, Springer, 2021.
[45]M. Tariq and K. Choi, ”YOLO11-driven deep learning approach for enhanced detection and visualization of wrist fractures in X-ray images,” *Mathematics*, vol. 13, no. 9, Art. no. 1419, 2025.
[46]F. Isensee, P. F. Jaeger, S. A. A. Kohl, J. Petersen, and K. H. Maier-Hein, ”nnU-Net: A self-configuring method for deep learning-based biomedical image segmentation,” *Nature Methods*, vol. 18, no. 2, pp. 203–211, 2021.
[47]A. Hatamizadeh, Y. Tang, V. Nath, D. Yang, A. Myronenko, B. Landman, H. R. Roth, and D. Xu, ”UNETR: Transformers for 3D Medical Image Segmentation,” *Proceedings of the IEEE/CVF Winter Conference on Applications of Computer Vision (WACV)*, pp. 574–584, 2022.
[48]Y. Pang, J. Liang, T. Huang, H. Chen, Y. Li, D. Li, L. Huang, and Q. Wang, ”Slim UNETR: Scale hybrid transformers to efficient 3D medical image segmentation under limited computational resources,” *IEEE Transactions on Medical Imaging*, vol. 43, no. 3, pp. 994–1005, 2023.
[49]L. Cao, Q. Zhang, C. Fan, and Y. Cao, ”Not Another Dual Attention UNet Transformer (NNDA-UNETR): A plug-and-play parallel dual attention block in U-Net with enhanced residual blocks for medical image segmentation,” *Quantitative Imaging in Medicine and Surgery*, vol. 14, no. 12, pp. 9169–9192, 2024.
[50]A. Alam, A. S. Al-Shamayleh, N. Thalji, A. Raza, E. A. Morales Barajas, E. B. Thompson, I. de la Torre Diez, and I. Ashraf, ”Novel transfer learning based bone fracture detection using radiographic images,” *BMC Medical Imaging*, vol. 25, no. 1, Art. no. 5, 2025.
[51]G. Deo, J. Totlani, and C. V. Mahamuni, ”A survey on bone fracture detection methods using image processing and artificial intelligence (AI) approaches,” in *AIP Conference Proceedings*, vol. 2802, no. 1, Art. no. 120022, 2024.
[52]S. Wang, J. Fei, Y. Liu, Y. Huang, and L. Li, ”Study on the application of deep learning artificial intelligence techniques in the diagnosis of nasal bone fracture,” *Int. J. Burns Trauma*, vol. 14, no. 6, pp. 125–132, 2024, doi: 10.62347/VCJP9652.
[53]S. A. Nadeem, A. P. Comellas, E. A. Regan, E. A. Hoffman, and P. K. Saha, ”Chest CT-based automated vertebral fracture assessment using artificial intelligence and morphologic features,” *Med. Phys.*, 2024, doi: 10.1002/mp.17072.
[54]Y. Gao, H. Chen, R. Ge, Z. Wu, H. Tang, D. Gao, X. Mai, L. Zhang, B. Yang, Y. Chen, and J. L. Coatrieux, ”Deep learning-based framework for segmentation of multiclass rib fractures in CT utilizing a multi-angle projection network,” *Int. J. Comput. Assist. Radiol. Surg.*, vol. 17, pp. 1115–1124, 2022, doi: 10.1007/s11548-022-02607-1.
[55]S. Turk, O. Bingol, A. Coskuncay, and T. Aydin, ”The impact of implementing backbone architectures on fracture segmentation in X-ray images,” *Eng. Sci. Technol., Int. J.*, vol. 59, p. 101883, 2024, doi: 10.1016/j.jestch.2024.101883.
[56]L. Oakden-Rayner, W. Gale, T. A. Bonham, M. P. Lungren, G. Carneiro, A. P. Bradley, et al., ”Validation and algorithmic audit of a deep learning system for the detection of proximal femoral fractures in patients in the emergency department: A diagnostic accuracy study,” *Lancet Digit. Health*, vol. 4, no. 5, pp. e351–e358, 2022, doi: 10.1016/S2589-7500(22)00004-8.
[57]L. Jin, J. Yang, K. Kuang, B. Ni, Y. Gao, Y. Sun, et al., ”Deep-learning-assisted detection and segmentation of rib fractures from CT scans: Development and validation of FracNet,” *eBioMedicine*, vol. 62, p. 103106, 2020, doi: 10.1016/j.ebiom.2020.103106.

[58]J. Olczak, F. Emilson, A. Razavian, T. Antonsson, A. Stark, and M. Gordon, ”Ankle fracture classification using deep learning: Automating detailed AO Foundation/Orthopedic Trauma Association (AO/OTA) 2018 malleolar fracture identification reaches a high degree of correct classification,” *Acta Orthop.*, vol. 92, no. 1, pp. 102–108, 2020, doi: 10.1080/17453674.2020.1837420.

[59]Z. Zhou, Z. Fu, J. Jia, and J. Lv, ”Rib fracture detection with dual-attention enhanced U-Net,” *Comput. Math. Methods Med.*, vol. 2022, p. 8945423, 2022, doi: 10.1155/2022/8945423.

[60]J. Schilcher, A. Nilsson, O. Andlid, and A. Eklund, ”Fusion of electronic health records and radiographic images for a multimodal deep learning prediction model of atypical femur fractures,” *Comput. Biol. Med.*, vol. 168, p. 107704, 2024, doi: 10.1016/j.compbiomed.2023.107704.

[61]E. Choi, D. Park, G. Son, S. Bak, T. Eo, D. Youn, and D. Hwang, ”Weakly supervised deep learning for diagnosis of multiple vertebral compression fractures in CT,” *Eur. Radiol.*, vol. 34, no. 6, pp. 3750–3760, 2024.

[62]K.-Y. Li, H.-B. Ye, Y.-L. Zhang, J.-W. Huang, H.-L. Li, and N.-F. Tian, ”Enhancing diagnostic accuracy of fresh vertebral compression fractures with deep learning models,” *Spine*, vol. 50, no. 16, pp. E330–E335, 2025.

[63]V. Marting, N. Borren, M. R. van Diepen, E. M. M. van Lieshout, M. E. Wijffels, and T. van Walsum, ”A deep learning-based approach to automated rib fracture detection and CWIS classification,” *Int. J. Comput. Assist. Radiol. Surg.*, vol. 20, no. 7, pp. 1381–1389, 2025.

[64]S. Kim, K. Shin, H.-S. Kim, Y. Kim, J. H. Kim, M. W. Joo, W. Kim, J. H. Park, Y. J. Cho, M. Kim, et al., ”Deep Learning Model for Differentiating Between Neoplastic Pathologic Fracture and Nonpathologic Fracture Using Hip Radiographs,” *JBJS*, pp. 10–2106, 2021.

[65]K. D. Oude Nijhuis, J. Prijs, B. Barvelink, H. van Luit, Y. Zhao, Z. Liao, R. L. Jaarsma, F. F. A. IJpma, M. E. Wijffels, J. N. Doornberg, et al., ”Open-source convolutional neural network to classify distal radial fractures according to the AO/OTA classification on plain radiographs,” *Eur. J. Trauma Emerg. Surg.*, vol. 51, no. 1, p. 261, 2025.

[66]J. Yan, H. Wang, X. Xu, S. Ji, Q. Lu, J. Zheng, Y. Ma, and R. Cao, ”MSDA-MLNet Based Cervicothoracic Junction Fracture Grading,” in *2025 13th Int. Conf. Adv. Cloud Big Data (CBD)*, 2025, pp. 68–73.

[67]C.-T. Cheng, L.-W. Kuo, C.-H. Ouyang, C.-P. Hsu, W.-C. Lin, C.-Y. Fu, S.-C. Kang, and C.-H. Liao, ”Development and evaluation of a deep learning-based model for simultaneous detection and localization of rib and clavicle fractures in trauma patients’ chest radiographs,” *Trauma Surg. Acute Care Open*, vol. 9, no. 1, 2024.

[68]J. Liu, P. Sun, Y. Yuan, Z. Chen, K. Tian, Q. Gao, X. Li, L. Xia, J. Zhang, and N. Xu, ”YOLOv12 algorithm-aided detection and classification of lateral malleolar avulsion fracture and subfibular ossicle based on CT images: Multicenter study,” *JMIR Med. Inform.*, vol. 13, p. e79064, 2025.

[69]A. Verma, V. Kumar, R. K. Yadav, et al., ”Humerus bone fracture detection utilizing YOLOv4 algorithm: A deep learning approach,” in *2024 2nd Int. Conf. Disruptive Technol. (ICDT)*, 2024, pp. 1191–1196.

[70]R. B. Nejad, A. H. Komijani, and E. Najafi, ”Intelligent cervical spine fracture detection using deep learning methods,” arXiv:2311.05708, 2023, doi: 10.48550/arXiv.2311.05708.

[71]N. Van der Gaast, P. Bagave, N. Assink, S. Broos, R. L. Jaarsma, M. J. R. Edwards, E. Hermans, F. F. A. IJpma, A. Y. Ding, J. N. Doornberg, et al., ”Deep learning for tibial plateau fracture detection and classification,” *Knee*, vol. 54, pp. 81–89, 2025.

[72]H. Kim, Y. D. Jeon, K. B. Park, H. Cha, M.-S. Kim, J. You, S.-W. Lee, S.-H. Shin, Y.-G. Chung, S. B. Kang, W. S. Jang, and D.-K. Yoon, ”Automatic segmentation of inconstant fractured fragments for tibia/fibula from CT images using deep learning,” *Sci. Rep.*, vol. 13, p. 20431, 2023, doi: 10.1038/s41598-023-47706-4.

[73]N. Ohs, C. J. Collins, D. C. Tourolle, P. R. Atkins, B. J. Schroeder, M. Blauth, P. Christen, and R. Müller, ”Automated segmentation of fractured distal radii by 3D geodesic active contouring of in vivo HR-pQCT images,” *Bone*, vol. 147, p. 115930, 2021, doi: 10.1016/j.bone.2021.115930.

[74]J. Chen, S. Liu, Y. Li, Z. Zhang, N. Liao, H. Shi, W. Hu, Y. Lin, Y. Chen, B. Gao, et al., ”Deep learning model for automated detection of fresh and old vertebral fractures on thoracolumbar CT,” *Eur. Spine J.*, vol. 34, no. 3, pp. 1177–1186, 2025.

[75]S. J. Nesheli, M. Sabet, A. Koozari, P. Mirzaghavami, A. Eftekhar, M. Elhaie, S. Rouhi, N. J. Lariche, M. Abidi, and S. M. Rezaeijo, ”Transformer-based and CNN-based models for clinically effective 2D and 3D pelvic bone segmentation in CT imaging,” *BMC Musculoskelet. Disord.*, vol. 27, no. 1, p. 68, 2026.

[76]S. Chakladar, D. E. Pereira, N. Tang, R. Siddabattula, and P. Hosseinzadeh, ”Development and Validation of an Automated Pipeline for the Detection of Monteggia Fracture Dislocations in Pediatric Radiographs,” *J. Pediatr. Orthop.*, pp. 10–1097, 2025.
[77]H. Chen, D. Dreizin, C. Gomez, A. Zapaishchykova, and M. Unberath, ”Interpretable severity scoring of pelvic trauma through automated fracture detection and Bayesian inference,” *IEEE Trans. Med. Imaging*, vol. 44, no. 1, pp. 130–141, 2024.
[78]C. Glessgen, J. Cyriac, S. Yang, S. Manneck, H. Wichtmann, A. M. Fischer, H.-C. Breit, and D. Harder, ”A deep learning pipeline for systematic and accurate vertebral fracture reporting in computed tomography,” *Clin. Radiol.*, vol. 83, p. 106827, 2025.
[79]J. Mongan, L. Moy, and C. E. Kahn Jr., ”Checklist for Artificial Intelligence in Medical Imaging (CLAIM): A Guide for Authors and Reviewers,” *Radiology: Artificial Intelligence*, vol. 2, no. 2, p. e200029, Mar. 2020, doi: 10.1148/ryai.2020200029.
[80]G. S. Collins, K. G. M. Moons, P. Dhiman, R. D. Riley, A. L. Beam, B. Van Calster, M. Ghassemi, X. Liu, J. B. Reitsma, M. van Smeden, *et al.*, ”TRIPOD+AI statement: Updated guidance for reporting clinical prediction models that use regression or machine learning methods,” *BMJ*, vol. 385, p. e078378, 2024, doi: 10.1136/bmj-2023-078378.
[81]B. Vasey, B. Nagendran, B. A. Campbell, D. Clifton, G. A. Collins, L. A. Denaxas, A. F. Gao, M. Liu, P. A. McCulloch, P. Moons, *et al.*, ”Reporting guideline for the early-stage clinical evaluation of decision support systems driven by artificial intelligence: DECIDE-AI,” *Nature Medicine*, vol. 28, no. 5, pp. 924–933, May 2022, doi: 10.1038/s41591-022-01772-9.
[82]A. Spångeus, T. Bjerner, M. Lindblom, C. Götz, A. Hummer, C. Salzlechner, and M. Woisetschläger, ”Breaking the silence: AI's contribution to detecting vertebral fractures in opportunistic CT scans in the elderly a validation study,” *Arch. Osteoporos.*, vol. 20, no. 1, p. 42, 2025.
[83]F. Atitallah, A. Thabet, J. C. Ayena, and N. Mezghani, ”Toward a unified public x-ray dataset integrating multiple databases to advance complex fracture analysis,” The Third International Conference on AI-Health (IARIA, AIhealth'26), pp. 75–81, March 8–12, Valencia, Spain, 2026.
[84]T. Greenhalgh, J. Wherton, C. Papoutsi, J. Lynch, G. Hughes, S. Hinder, N. Fahy, R. Procter, S. Shaw, *et al.*, ”Beyond adoption: A new framework for theorizing and evaluating nonadoption, abandonment, and challenges to the scale-up, spread, and sustainability of health and care technologies,” *J. Med. Internet Res.*, vol. 19, no. 11, p. e8775, 2017.
[85]J. R. Zech, S. M. Santomartino, and P. H. Yi, ”Artificial intelligence (AI) for fracture diagnosis: An overview of current products and considerations for clinical adoption, from the AJR special series on AI applications,” *Am. J. Roentgenol.*, vol. 219, no. 6, pp. 869–878, 2022.
[86]M. K. Elbahi, A. Muhammed, M. F. A. Mohamednour, and F. S. Mukhtar, ”Artificial Intelligence in Fracture Diagnosis on Radiographs: Evidence, Pitfalls, and Pathways for Clinical Integration (2020–2025),” *Cureus*, vol. 17, no. 9, 2025.
[87]M. M. Chen, L. P. Golding, and G. N. Nicola, ”Who Will Pay for AI?,” *Radiol. Artif. Intell.*, vol. 3, no. 3, p. e210030, 2021.
[88]J. Amann, A. Blasimme, E. Vayena, D. Frey, V. I. Madai, and the Precise4Q Consortium, ”Explainability for artificial intelligence in healthcare: A multidisciplinary perspective,” *BMC Med. Inform. Decis. Mak.*, vol. 20, no. 1, p. 310, 2020.
[89]A. Kebaili, J. Lapuyade-Lahorgue, and S. Ruan, ”Deep learning approaches for data augmentation in medical imaging: a review,” *J. Imaging*, vol. 9, no. 4, p. 81, 2023.
[90]S. Vironicka and J. G. R. Sathiaseelan, ”Classification of Long-Bone Fractures Using Modified Faster R-CNN for X-Ray Images,” *Indian Journal of Science and Technology*, vol. 16, no. 1, pp. 56–65, 2023.
[91]J. Bae, S. Yu, J. Oh, T. H. Kim, J. H. Chung, H. Byun, M. S. Yoon, C. Ahn, and D. K. Lee, ”External validation of deep learning algorithm for detecting and visualizing femoral neck fracture including displaced and non-displaced fracture on plain X-ray,” *Journal of Digital Imaging*, vol. 34, no. 5, pp. 1099–1109, 2021.
[92]C. Blüthgen, A. S. Becker, I. V. de Martini, A. Meier, K. Martini, and T. Frauenfelder, ”Detection and localization of distal radius fractures: Deep learning system versus radiologists,” *European Journal of Radiology*, vol. 126, p. 108925, 2020.
